# A methodology for classifying tissue-specific metabolic and inflammatory receptor functions applied to subcutaneous and visceral adipose

**DOI:** 10.1371/journal.pone.0276699

**Published:** 2022-10-25

**Authors:** Gur Arye Yehuda, Judith Somekh

**Affiliations:** Information Systems, University of Haifa, Haifa, Israel; Valahia University of Targoviste: Universitatea Valahia din Targoviste, ROMANIA

## Abstract

To achieve homeostasis, the human biological system relies on the interaction between organs through the binding of ligands secreted from source organs to receptors located on destination organs. Currently, the changing roles that receptors perform in tissues are only partially understood. Recently, a methodology based on receptor co-expression patterns to classify their tissue-specific metabolic functions was suggested. Here we present an advanced framework to predict an additional class of inflammatory receptors that use a feature space of biological pathway enrichment analysis scores of co-expression networks and their eigengene correlations. These are fed into three machine learning classifiers–eXtreme Gradient Boosting (XGBoost), Support Vector Machines (SVM), and K-Nearest Neighbors (k-NN). We applied our methodology to subcutaneous and visceral adipose gene expression datasets derived from the GTEx (Genotype-Tissue Expression) project and compared the predictions. The XGBoost model demonstrated the best performance in predicting the pre-labeled receptors, with an accuracy of 0.89/0.8 in subcutaneous/visceral adipose. We analyzed ~700 receptors to predict eight new metabolic and 15 new inflammatory functions of receptors and four new metabolic functions for known inflammatory receptors in both adipose tissues. We cross-referenced multiple predictions using the published literature. Our results establish a picture of the changing functions of receptors for two adipose tissues that can be beneficial for drug development.

## Introduction

As the human system instinctively and continuously aims to maintain a steady state, the biological system reacts to different conditions by activating feedback control loops between the cells in tissues, which is manifested through the binding of chemical structures called receptors to their ligands [1]. These receptors are proteins, usually cell surface receptors, which bind to their ligands and cause a required response in the cell. When a ligand binds to its corresponding receptor, it activates or inhibits the receptor’s associated biochemical pathway. Receptors can control membrane channels, induce cell growth, division, and death [1]. For example, insulin is a metabolic hormone ligand that is secreted from pancreatic cells into the bloodstream to bind distant insulin receptors located on various cell types [2]. Upon insulin binding, the insulin receptors start a cascade of molecular events that result in, among other developments, glucose absorption by the cells [3]. Another example is cytokines, which are ligands that serve as immunomodulating agents [4]. As such, they have immune-signaling and inflammatory receptors that respond to circulating levels of proinflammatory cytokines, adipokines and other immune markers and trigger immune and inflammatory signaling pathways that are found in various cell types, including immune cells and non-immune cells[5].

Since receptors play an important role in signal transduction within the cell, many drugs are designed to target receptors [6,7] and understanding the functions these receptors fulfill in different tissues is crucial in the development of these drugs. Despite years of biological experimental research, the current knowledge and understanding of the functions in general and the tissue-specific functions of many receptors specifically are lacking.

High-throughput sequencing technologies generate gene expression data that measure the expression level of thousands of genes from a single experiment. Today, these technologies and algorithmic advancements enable us to research simultaneously hundreds of genes coded to receptors. A common task of gene expression analysis is the detection of gene–gene co-expression networks. The most popular method for specifying co-expression networks is Weighted Gene Co-Expression Network Analysis (WGCNA) [8]. The WGCNA algorithm gathers together into gene modules (networks) related genes based on their co-expression patterns and topological closeness to neighbor genes in the network. The main concept behind WGCNA is that genes with similar functions might be co-expressed [9] and thus co-expression networks are used to identify and categorize the functional roles of genes whose function is unknown.

Supervised learning methods have been used to predict protein functions from gene expression data and gene co-expression [10–17]. For example, Support Vector Machines (SVMs) have successfully classified functional modules and protein interaction networks from gene expression data [18]. Brown et al. [10] proposed a method for functionally categorizing genes based on gene expression data. The authors examined numerous SVM models as well as other supervised learning approaches such as Parzen windows (a nonparametric method for estimating continuous density function), Fisher’s linear discriminant analysis (LDA), two decision tree classifiers (C4.5 and MOC1), and SVMs with different kernels. They discovered that SVM models with a radial kernel were the best at identifying groupings of genes with a common function using expression data according to the cost function the authors defined, trying to minimize the false-positive (FP) and false-negative (FN) errors (when giving double weight to the FN mistakes). The authors used log-transformed gene expression levels, i.e., DNA microarray hybridization experiments data, as the features’ space for the SVM models and measured their performances compared to the null learning producer that classifies all test examples as negative (a dummy classifier that always predicts “negative” for all examples). The results showed that SVM can recognize some functional classes successfully and outperformed the other examined models. Furthermore, based on their expression data, they employed SVMs to infer new functional functions for unannotated yeast genes.

Kiliç et al. [11] tested the SVM model for semi-supervised positive unlabeled (PU) learning (further discussed in the Methods section) as part of a survey of PU learning algorithms. The authors used Escherichia coli gene expression data combined with known protein interactions in such a way that if two proteins are known to be interacting, the example consisting of their expression profiles is a positive example and all other protein pairs are treated as (unlabeled) negative examples. SVM showed promising results for protein interaction predictions. Although clusters of gene expression profiles can be informative about function, they might not always be coherent, as pointed out by Zhou et al [12]. The latter authors investigated a graph-theoretic approach, in which genes are encoded as nodes, and edges connect genes with correlated expression profiles—a co-expression network. They carry on conducting a simple experiment in which the shortest path between genes with the same GO (gene ontology) term is analyzed to determine whether genes in the path belong to the same GO term or GO terms that are ancestors or descendants in the ontology. Wu et al. [13] showed that this method can be used to predict the function of unknown genes from known genes that are part of the same shortest path with good accuracy for several types of genes (mitochondrial and cytoplasmic) but with medium accuracy for nuclear genes. Romero et al. [14] proposed a method that combines cluster analysis with hierarchical multi-label classification (HMC) in which examples may belong to more than one class at each hierarchical level at the same time. They employed spectral clustering to extract novel features from the gene co-expression network (GCN) to enhance the function prediction job. To generate consistent predictions, they emphasized the need to develop new characteristics that indicate the GCN structural qualities and the hierarchical structure of biological processes. Obregón et al. [15] used the gene’s location in the genomes to which they belong to predict their function. They executed machine learning models and trained them using attributes derived from the location of genes in the genomes to which they belong to predict thousands of gene functions. The authors demonstrated that, in some situations, gene location alone can be more valuable than sequencing in determining gene function. Peng et al. [16] used network correlation to create a semi-supervised autoencoder approach for integrating various networks and generating a low-dimensional feature representation. The authors used multi-network embedding using a semi-Auto Encoder to map input networks into a non-linear and low-dimension space. A convolutional neural network based on those integrated features’ embeddings was used to identify unlabeled gene functions. Both yeast and human datasets were evaluated, and the approach outperformed three other methods. Tahzeeb et al. [17] examined the ability of several neural networks to predict protein function using Gene Ontology terms. Each protein instance was associated with several Gene Ontology (GO) terms of molecular function, resulting in a multilabel classification of protein functions using a dataset of reviewed protein entries from nine bacterial phyla. In addition to the association of each protein to multiple terms of GO molecular function, the dataset includes features such as the sequence of amino acids that make up the corresponding protein, compositions of amino acids, dipeptides, and tripeptides; compositions of five groups of amino acids, namely aliphatic, aromatic, positively charged, negatively charged, and uncharged, and various structural and physiochemical properties derived from the amino acid sequence. The researchers found that single-layer neural networks with a small number of neurons outperformed multi-layer neural networks.

The GTEx project [19] includes a unique collection of more than 8000 samples of RNA-seq gene expression data across multiple tissues collected from ~1000 donors. Using this data and focusing on metabolic and inflammatory roles of receptors, we ask the following question: How can we use gene expression data to predict the function of genes corresponding to proteins that represent receptors? Specifically, we focus on predicting two receptor functions: (1) metabolic functions that are related to the metabolic/endocytosis/growth regulation systems [20–22] and trigger various metabolic signaling pathways within the cell and (2) inflammatory functions that respond to circulating levels of proinflammatory cytokines, adipokines and other inflammatory markers and trigger inflammatory signaling pathways within the cell.

Somekh [23] suggested an approach for predicting the tissue-specific metabolic functions of receptor proteins based on gene expression data. The method was based on detecting receptor expression coordination patterns for over 700 receptors and predicting the metabolic roles of receptors in subcutaneous adipose tissue. The enrichment analysis scores of the receptor’s co-expression networks were fed as an input to SVM and k-NN classifiers. Using a semi-supervised technique and literature survey, Somekh [23] compiled a list of known metabolic and non-metabolic receptors. Pathway enrichment scores were found by the authors to be highly successful indicators of correctly categorizing metabolic receptors in the subcutaneous adipose tissue.

Here we extend and refine this previous work [23] that predicted metabolic receptors in adipose subcutaneous by offering (1) an additional class of inflammatory receptors to classify three receptor classes–“metabolic”, “inflammatory”, and “other” class (neither metabolic nor inflammation-related), (2) an additional visceral adipose tissue, (3) an additional machine learning model–the XGBoost, and (4) a new feature for each tested receptor, based on the correlations between the receptor’s composing co-expression module eigengene and the correlations between this eigengene and the rest of the co-expression modules’ eigengenes. We add this feature to account for the modules’ connectivity, e.g., to include data on “close” metabolic modules that might be positively correlated and may add more knowledge on receptor roles using its co-expression.

## Results

**Fig 1:**
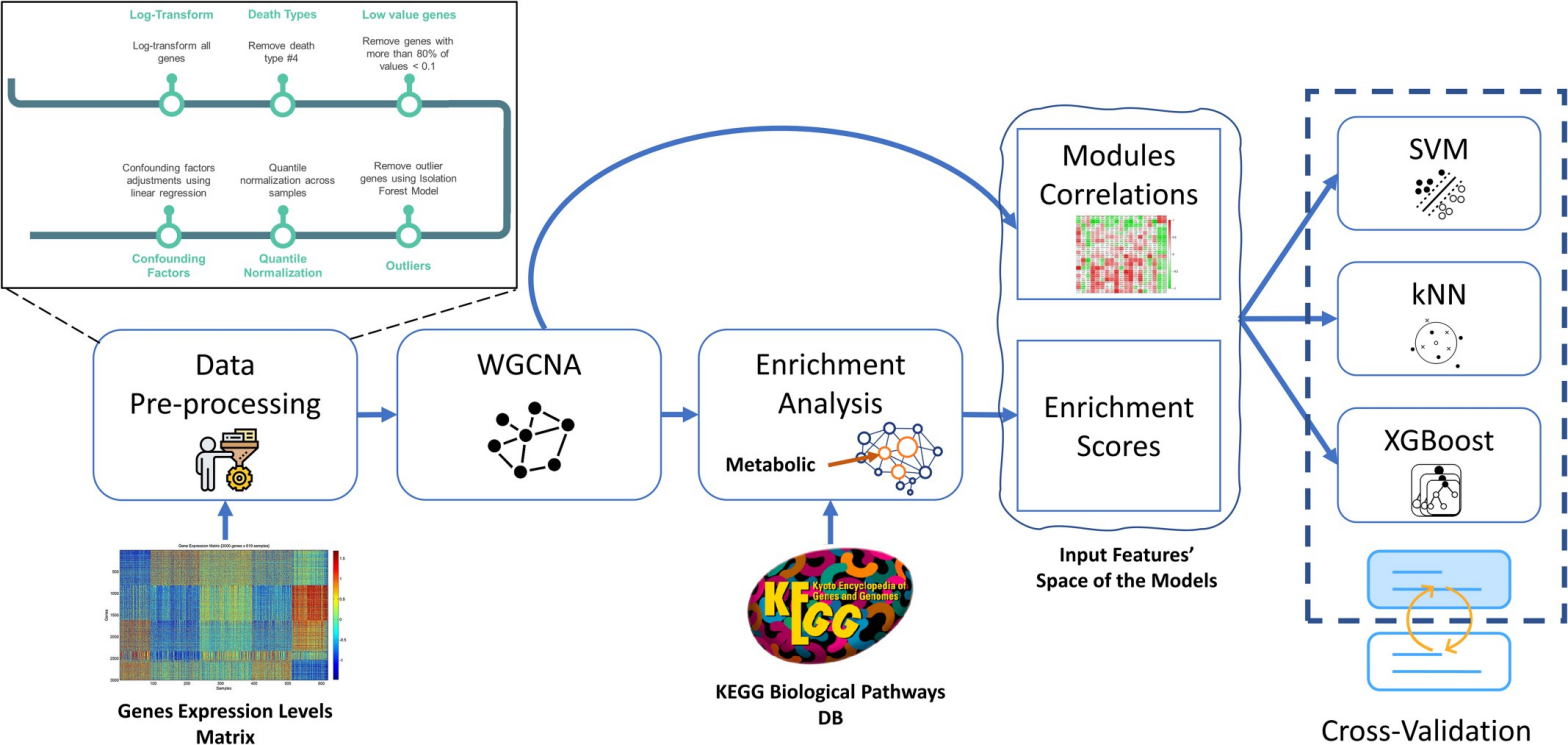
Schematic workflow of data preprocessing and the proposed methodology.

Our methodology classifies three classes of receptors applying to two adipose tissues. We validated our approach on the known labeled tissue-specific functions of receptors and further used our approach to predict new tissue-specific “metabolic”, “inflammatory”, and “other” functions of receptors in subcutaneous and visceral adipose. Our methodology is detailed in the following sections and a schematic view is presented in Fig 1.

### Receptor labeled lists

To construct a machine learning model, a labeled list of known receptor classes in each tissue is required. As mentioned above, we focused on three classes of receptors–“metabolic”, “inflammatory” and “other”. By “metabolic” we refer to receptors related to the metabolic, endocytosis, or growth regulation systems [20–22]. By “inflammatory” we refer to receptors that are activated as a result of immune-related stimuli such as inflammation or chronic disease by known immune-related ligands such as cytokines and chemokines. By “other” we refer to a group of receptors that are neither metabolic nor inflammatory.

Receptor labeling was challenging since tissue-specific labeling of receptors is not an established knowledge and we had to generate it. For the classification of metabolic receptors, we used the “metabolic” labeling created by Somekh et al. [23], which was based on a literature review and semi-supervised learning. We generated the “inflammatory” labeling using the known cytokine receptors derived from the KEGG (Kyoto Encyclopedia of Genes and Genomes) [24] database, the “cytokine-cytokine receptor interaction” KEGG pathway. The “other” class was inferred using semi-supervised learning (see details in the Methods section). Examples of receptors that were labeled as "other" are—GFRA2, HTR1F, KCNA3, ADCY7 and CATSPER1. GFRA2 gene encodes to a potent neurotropic factor and a receptor of Neurturin (NRTN) which regulate the survival and function of neurons [“GFRA2 GDNF family receptor alpha 2 [Homo sapiens (human)]”. NCBI. Retrieved 21 July 2022]. HTR1F gene encodes serotonin 5-TH 1F receptor that bind to the endogenous neurotransmitter serotonin and mediate inhibitory neurotransmission ["HTR2C 5-hydroxytryptamine receptor 2C [Homo sapiens (human)]". NCBI. Retrieved 21 July 2022]. KCNA3 gene encodes the Potassium voltage-gated channel, shaker-related subfamily, member 3 protein. Potassium channels represent the most complex class of voltage-gated ion channels from both functional and structural standpoints. Their diverse functions include regulating neurotransmitter release, heart rate, insulin secretion, neuronal excitability, epithelial electrolyte transport, smooth muscle contraction, and cell volume [“KCNA3 potassium voltage-gated channel subfamily A member 3 [Homo sapiens (human)]”, NCBI. Retrieved 21 July 2022]. Adenylate Cyclase 7 (ADCY7) encodes a membrane-bound adenylate cyclase that catalyzes the formation of cyclic AMP from ATP and is inhibitable by calcium [“ADCY7 adenylate cyclase 7 [ Homo sapiens (human)]”. NCBI. Retrieved 21 July 2022]. The Cation Channel Sperm Associated 1 (CATSPER1) plays a central role in calcium-dependent physiological responses essential for successful fertilization, such as sperm hyperactivation, acrosome reaction and chemotaxis towards the oocyte [“CATSPER1 Cation Channel Sperm Associated 1 [Homo sapiens (human)]”. NCBI. Retrieved 21 July 2022].

This way we labeled three known receptor classes: “metabolic”, “inflammatory” and “other”. After labelling and processing, we had 44 “metabolic” receptors, 40 “inflammatory” receptors, and 50 “other” receptors for subcutaneous adipose and 45 “metabolic” receptors, 47 “inflammatory” receptors, and 48 “other” receptors for visceral adipose.

### Data preparation

The GTEx subcutaneous and visceral adipose gene expression data were filtered, pre-processed, and corrected for batch effects (see the Methods section). After filtering, we were left with 656 samples and 16,058 genes for subcutaneous adipose and 486 samples, and 16,091 genes for visceral adipose.

### Co‑expression module construction and annotation

**Fig 2:**
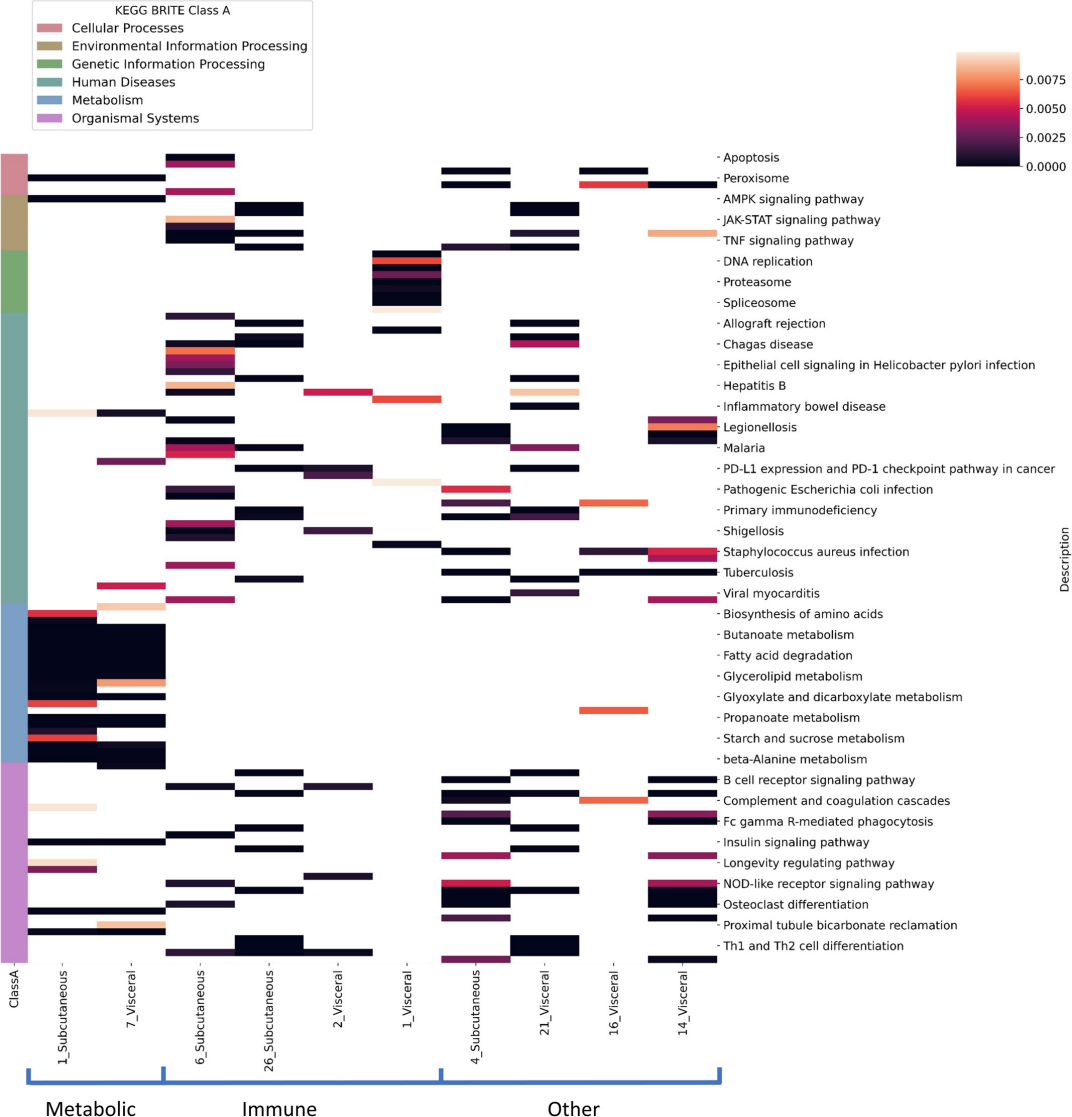
Pathway enrichment analysis of ten representative WGCNA modules. The significantly enriched KEGG pathways (adjusted p-values < = 0.01) and enrichment scores calculated for subcutaneous and visceral adipose are presented. The columns present the modules (that include many labeled receptors) in each tissue and the rows represent the significantly enriched KEGG biological pathways. The matrix cells present the enrichment scores of the pathways for each module. The pathways are classified into six classes according to the BRITE classification and highlighted in the annotation column to the left (see the y-axis). For example, you can see that the modules that include many metabolic receptors (highlighted as “Metabolic” on the x-axis) are highly enriched with the “Metabolism” classification (colored in blue on the y-axis).

We utilized the WGCNA [8] algorithm to generate 61 subcutaneous adipose co-expression networks and 38 visceral adipose co-expression networks (see Methods section). Module (cluster) dendrograms for subcutaneous and visceral adipose can be found in S3 and S4 Figs in S1 File, respectively. Following the construction of the modules, we executed KEGG pathway enrichment analysis for each module to generate their enrichment scores [25]. The annotations of modules that include multiple known labeled receptors are demonstrated in Fig 2. The figure presents a heatmap of ten representative WGCNA co-expression modules and their enrichment scores (-log(p-value) for p-values < 0.01) for KEGG’s biological pathways for both subcutaneous and visceral adipose. It can be seen that the modules that are enriched with multiple known metabolic receptors (highlighted as “Metabolic” on the x-axis), are enriched with metabolic biological pathways. For example, module #1 in subcutaneous adipose, which includes 52% of our “metabolic” labeled receptors (see S4 Table in S1 File), is highly enriched with metabolic KEGG pathways that are classified as a “Metabolism” class according to the BRITE classification (shown in blue on the annotation column to the left). The modules that include multiple inflammatory receptors (highlighted as “Immune” on the x-axis) are significantly enriched with multiple pathways that are classified as “Human Diseases” (highlighted in green on the annotation column to the left). Heatmaps that include the full list of generated modules and their significantly enriched pathways are presented in S1 and S2 Figs in S1 File for subcutaneous and visceral adipose, respectively. S4 and S5 Tables in S1 File show that many receptors with similar functions tend to be clustered together across several main modules and present the percentages of labeled receptors from each class within the WGCNA modules. The full distribution of labeled receptors into the different WCGNA-generated modules can be found in S7 Table in S1 File. Nevertheless, there are receptors, e.g., metabolic receptors, that are clustered in distinct modules and can be detected as metabolic only by using the new feature of module correlations and enrichment scores that are fed into the machine learning classifiers.

### Machine learning model construction and validation

We employed the XGBoost, linear SVM, and k-NN models to tackle the problem of multiclass classification of receptors in subcutaneous and visceral adipose tissues (see the Methods section). All models utilized the following feature space per receptor: (1) the enrichment scores of the KEGG pathways applied to each receptor’s module, and (2) the receptor’s module eigengene correlations with other modules. To assess the performance of our classifiers, we utilized tenfold cross-validation (see the Methods section).

Table 1 shows the performance of the classifiers for each adipose tissue in the three-class experiment. It can be seen that the XGB classifier outperforms the SVM and k-NN classifiers for both tissues, with accuracies of 0.89 and 0.8 for subcutaneous adipose and visceral adipose, correspondingly.

**Table 1:** Performance evaluation for predicting “inflammatory”, “metabolic” and “other” receptor types in subcutaneous and visceral adipose.

	Method	Adipose tissue	Accuracy	Precision	Recall	F1
1	**XGB**	**Subcutaneous**	**0.89**	**0.91**	**0.88**	**0.87**
2	SVM	Subcutaneous	0.85	0.85	0.84	0.82
3	k-NN	Subcutaneous	0.87	0.89	0.87	0.86
1	**XGB**	**Visceral**	**0.8**	**0.83**	**0.8**	**0.79**
2	SVM	Visceral	0.69	0.74	0.69	0.66
3	k-NN	Visceral	0.79	0.81	0.79	0.78

We then investigated the receptors that were misclassified by our models, i.e., their known functions and the predicted functions were not identical. Table 2 presents the FP and FN misclassified receptors and highlights in bold the common misclassified receptors in both tissues. Interestingly, the EPOR, TNFRSF21, TNFRSF25, and IFNGR1 receptors that we labeled as inflammatory (based on KEGG’s cytokine-cytokine receptor interaction pathway) are predicted to be “metabolic” for both adipose tissues. LDLR and TFRC that we labeled as metabolic are both predicted to be “inflammatory” by the model for both adipose tissues. Some of the predictions are consistent for both tissues. For example, the inflammatory labeled receptors EPOR, TNFRSF21, IFNGR1, and TNFRSF25, as noted before, are predicted by the models to be metabolic (and not “other”) in both tissues. Indeed, we found experimental validation supporting our predictions (misclassifications) which we elaborate on in the discussion section.

**Table 2:** Misclassified receptors in subcutaneous and visceral adipose with their true/predicted labels and probabilities.

	Gene Symbol	Tissue	True Label	Predicted Label	inflammatory probability	Metabolic probability	Other probability
1	LTBR	SA	inflammatory	Metabolic	0.027	0.953	0.019
**2**	**EPOR**	SA/VA	Inflammatory	Metabolic	0.014/0.013	0.982/0.982	0.004/0.005
**3**	**TNFRSF25**	SA/VA	Inflammatory	Metabolic	0.140/0.124	0.843/0.867	0.017/0.009
**4**	**TNFRSF21**	SA/VA	Inflammatory	Metabolic	0.004/0.008	0.992/0.989	0.004/0.004
5	IL17RA	SA	Inflammatory	Metabolic	0.026	0.968	0.006
6	IFNAR1	SA	Inflammatory	Metabolic	0.039	0.947	0.014
**7**	**IFNGR1**	SA/VA	Inflammatory	Metabolic	0.022/0.030	0.973/0.968	0.005/0.001
**8**	**CCR2**	SA/VA	Inflammatory	Other	0.010/0.014	0.003/0.002	0.987/0.984
9	XCR1	SA	Inflammatory	Other	0.157	0.012	0.831
**10**	**IL10RA**	SA/VA	Inflammatory	Other	0.032/0.003	0.010/0.001	0.958/0.996
**11**	**IL12RB1**	SA/VA	Inflammatory	Other	0.005/0.005	0.007/0.001	0.987/0.995
**12**	**IL2RG**	SA/VA	Inflammatory	Other	0.004/0.014	0.004/0.001	0.993/0.986
13	F3	SA	Metabolic	Inflammatory	0.919	0.076	0.004
**14**	**TFRC**	SA/VA	Metabolic	Inflammatory	0.603/0.776	0.340/0.215	0.057/0.009
**15**	**LDLR**	SA/VA	Metabolic	Inflammatory	0.877/0.986	0.071/0.008	0.052/0.007
16	LEPR	SA	Metabolic	Inflammatory	0.897	0.097	0.005
17	DRD4	SA	Metabolic	Inflammatory	0.962	0.027	0.010
18	ADRA2B	SA	Metabolic	Other	0.061	0.129	0.810
19	TFR2	SA	Metabolic	Other	0.011	0.002	0.987
20	CD28	SA	Other	Inflammatory	0.704	0.014	0.282
21	MPL	VA	Inflammatory	Metabolic	0.019	0.979	0.002
22	TNFRSF10D	VA	Inflammatory	Metabolic	0.133	0.856	0.012
23	FAS	VA	Inflammatory	Metabolic	0.061	0.934	0.005
24	IL22RA1	VA	Inflammatory	Metabolic	0.435	0.559	0.006
25	IL3RA	VA	Inflammatory	Metabolic	0.028	0.970	0.003
26	CD27	VA	Inflammatory	Other	0.115	0.001	0.884
27	CSF2RB	VA	Inflammatory	Other	0.035	0.002	0.963
28	CCR6	VA	Inflammatory	Other	0.211	0.008	0.781
28	IL2RB	VA	Inflammatory	Other	0.110	0.007	0.883
30	IL10RB	VA	Inflammatory	Other	0.004	0.001	0.995
31	EDNRB	VA	Metabolic	Inflammatory	0.970	0.025	0.005
32	NOTCH4	VA	Metabolic	Inflammatory	0.732	0.263	0.005
33	ADRB2	VA	Metabolic	Inflammatory	0.815	0.177	0.008
34	FGFR2	VA	Metabolic	Inflammatory	0.865	0.132	0.003
35	S1PR4	VA	Metabolic	Inflammatory	0.988	0.007	0.005
36	CATSPER1	VA	Other	Inflammatory	0.573	0.006	0.421
37	KCNN4	VA	Other	Inflammatory	0.660	0.004	0.336
38	CD5	VA	Other	Inflammatory	0.664	0.004	0.332
39	NCR3	VA	Other	Inflammatory	0.804	0.014	0.182
40	CD48	VA	Other	Inflammatory	0.503	0.013	0.484
41	ITGAL	VA	Other	Inflammatory	0.900	0.008	0.092

Receptors predicted as FN/FP in both adipose tissues are shown in bold. “SA” represents subcutaneous adipose and “VA” visceral adipose.

### Feature analysis for detecting significant biological pathways

**Fig 3:**
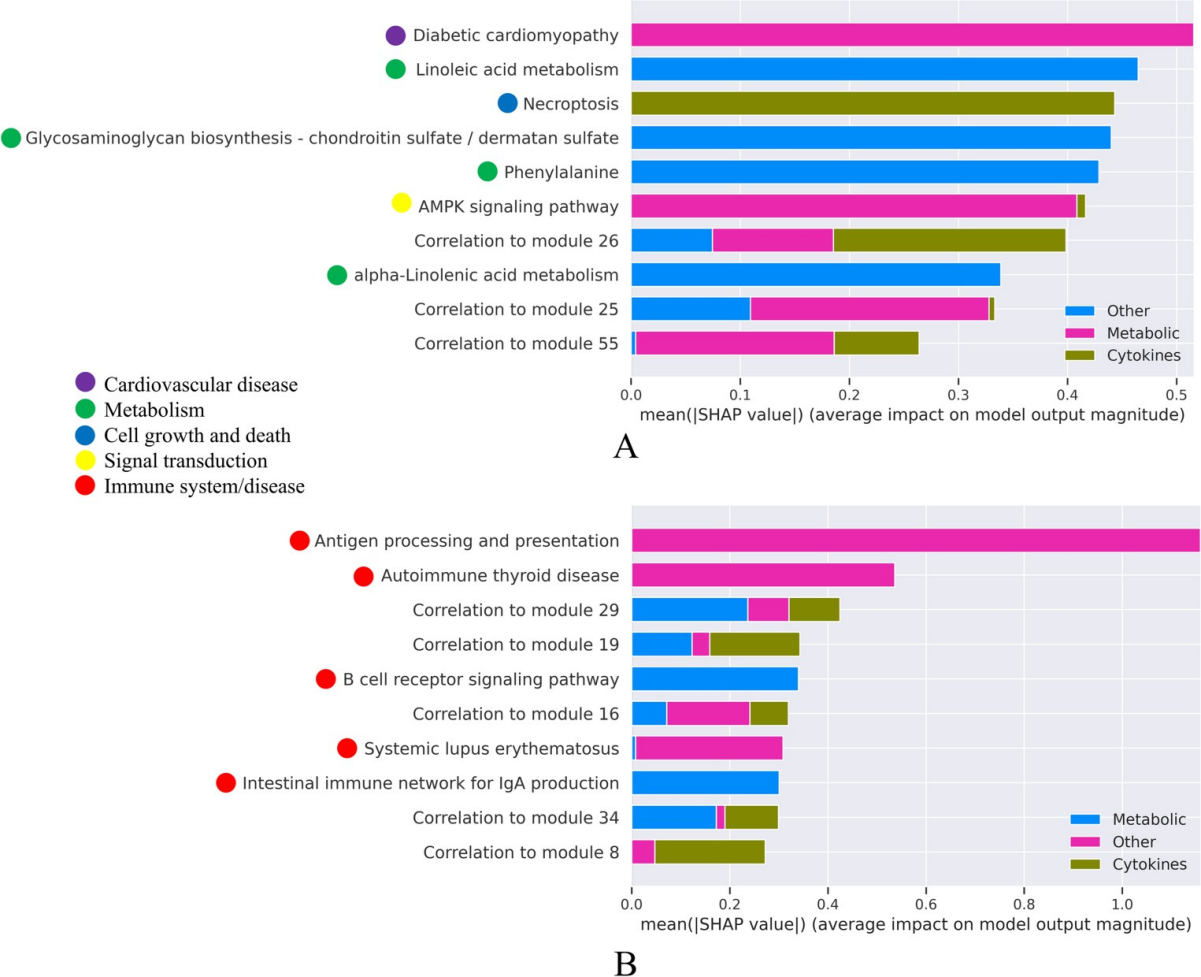
Top 10 features (pathways) and their average SHAP influence (absolute values) on the magnitude of the model prediction. A. Calculated for subcutaneous adipose. B. Calculated for visceral adipose. The SHAP method illustrates the magnitude of the effect of each feature (KEGG pathway) on each classification. The colored dots to the left highlight KEGG’s BRITE annotations of the pathways.

**Fig 4:**
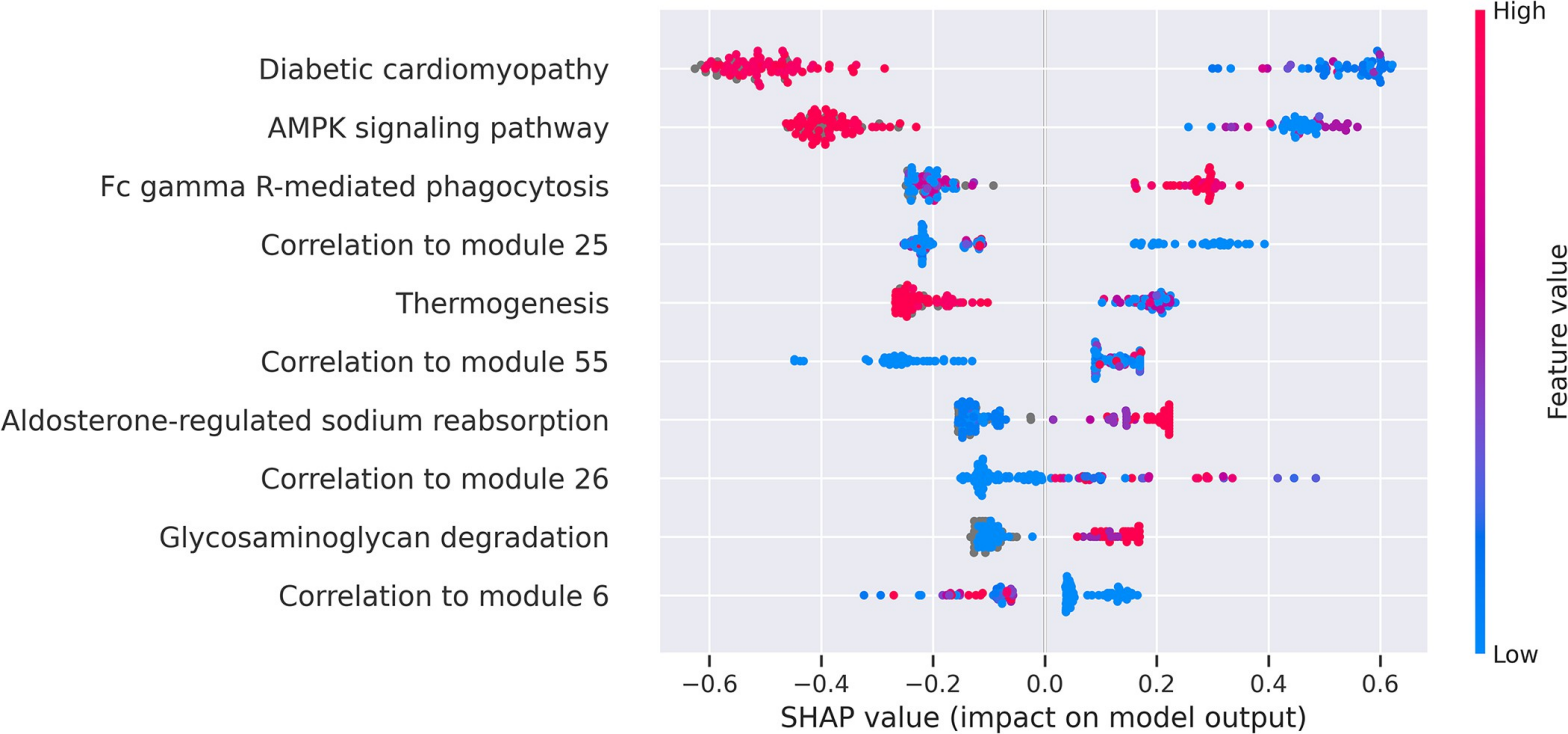
SHAP subcutaneous adipose “metabolic” class variable significance plot. The values show the impact of the feature on model output (prediction). The plot is composed of all receptors in the training data. SHAP values indicate how much the feature contributes to the classification.

We used feature analysis with the SHapley Additive exPlanations (SHAP) [26] to find the most predictive features, i.e., KEGG pathways significantly enriched with genes that are included in the receptor’s module and that drive the prediction of receptors (see the full description in the Experimental Design section). The SHAP values of each feature (KEGG pathway) represent the feature’s impact on the model output/classification of receptors. Fig 3A and 3B show the ten most important features for subcutaneous and visceral adipose tissues, respectively. It shows the average SHAP influence on the magnitude of model output in absolute values for the top ten features in our three-class model. It can be seen that the most important feature affecting the “metabolic” classification of receptors (highlighted in pink in Fig 3A) is the “Diabetic cardiomyopathy” pathway. Diabetic cardiomyopathy is defined as left ventricular dysfunction that occurs among patients with diabetes mellitus independent of a recognized cause such as coronary artery disease or hypertension [https://www.kegg.jp/entry/hsa05415] and is characterized by insulin and metabolic resistance genes [27]. The enrichment score of the “Necroptosis” pathway (third from the top in Fig 3A), which is related to cell apoptosis and death, is most significant for classifying inflammatory receptors. We also highlighted KEGG’s BRITE hierarchy for annotation of these top KEGG pathways. It can be seen that many of the top pathways that drive the classification of metabolic/inflammatory/other receptors are annotated as “Metabolism” (shown using green dots) and “Immune system/disease” (shown using red dots). For example, see Fig 3A where the “Linoleic acid metabolism”, “Glycosaminoglycan biosynthesis chondroitin sulfate”, “Phenylalanine” and “alpha-Linoleic acid metabolism” pathways are annotated as “Metabolism” (colored in green) for subcutaneous adipose and Fig 3B where the top five “Immune system/disease” annotated pathways for adipose visceral are shown in red. We note that the values are absolute and the interpretation is not always straightforward, meaning that the combination of distinct features between the three classes and the metabolically annotated pathways (highlighted in green) are presented by the model as important features to distinguish the “other” type of receptors from the “metabolic” and “inflammatory” classifications. To get a better understanding of what we were seeing, we used a directional SHAP analysis that can only be generated for two types of classes (since it shows direction). We analyzed the metabolic against the inflammatory classes and used the SHAP method to investigate the direction of each feature’s contribution to the class classification in subcutaneous adipose (see Fig 4).

Fig 4 shows the total magnitudes of the SHAP values over all samples as a plot of features sorted in descending order by their relevance and uses the SHAP values to highlight the distribution of their impact on the model output (metabolic) prediction. Here we analyze and show how the value of the feature affects the “metabolic” class as opposed to the “inflammatory” class. The horizontal position indicates the influence of each feature, i.e., whether that value’s effect is related to a greater or lower prediction for the metabolic class. The coloring corresponds to each feature’s original values across samples and indicates whether that feature value (pathway enrichment score) is high (red) or low (blue) for that observation. The SHAP values of each feature are represented on the x-axis and represent the feature’s impact on the model output; the features (e.g., KEGG pathways) are on the y-axis. For example, a high value (red colored dots) of the enrichment score for “Diabetic cardiomyopathy” (the third from the bottom) has a negative impact (a negative SHAP value on the x-axis) on the “metabolic” type receptor prediction. In other words, a higher enrichment score for this pathway drives a metabolic prediction in most cases, increasing the probability of the receptor being categorized as belonging to the metabolic receptor group. An additional analysis of the effect (direction) of each feature’s contribution to the metabolic, inflammatory or other class classification is presented in S5 and S6 Figs in S1 File.

### Prediction

Finally, we chose the XGB model, which outperformed the other models, to use for predicting the unlabeled receptors. Out of the 692 known receptors list derived from Ramilowski et al. [28] we retained the receptors that were included in the GTEx dataset, 594 and 600 for subcutaneous and visceral adipose respectively. From these, we retained the receptors that were included in the co-expression modules, 446 and 485 for subcutaneous and visceral adipose, respectively. We used 134 and 140 labeled receptors, for subcutaneous and visceral adipose respectively, for the training and testing phases (see methods). Finally, we executed the model to predict the function of the remaining 312 and 345 unlabeled receptors for subcutaneous and visceral adipose respectively. The XGB model predicted 96 and 46 new unknown inflammatory receptors and 24 and 22 new unknown metabolic receptors (with probability > 0.85) for subcutaneous and visceral adipose, respectively. These full lists of newly predicted receptors for subcutaneous and visceral adipose can be found in S1 and S2 Tables in S1 File, respectively. The receptors that were classified in the same way in both adipose tissues (classification probability > 0.85) are presented in Table 3. We surveyed the literature for relevant wet lab experiments in support of our predictions. Column 5 in Table 3 include the literature verification of the predictions, describing the experimental summary and the manuscript reference. We note that several inflammatory receptors (e.g., TNFRSF1B in row 9, Table 3) which were included in the KEGG “Cytokine-cytokine signaling” pathway, were previously filtered out by us (see Methods) since they are related to metabolic functions by GO. Nevertheless, these receptors are predicted by our classifiers to trigger inflammatory functions in adipose tissues. In addition, receptors that are strongly predicted to change functions (the predictions’ probability absolute difference is > 0.85) between the two tissues (e.g., predicted to be “metabolic” in one tissue and “inflammatory” in the other) are presented in S6 Table in S1 File.

**Table 3:** Receptors with unknown metabolic and inflammatory functions, which we predicted to have similar functions in both subcutaneous and visceral adipose tissues, and references supporting our predictions. Eight first receptors predicted to be “metabolic” are highlighted in light green and 15 “inflammatory” receptors are highlighted in red.

	Receptor	Class prob. SA	Class prob. VA	Summary of the experiment/s supporting our prediction and its literature reference
1	ABCA1	0.875	0.909	ABCA1 in adipocytes regulates adipose tissue lipid content, glucose tolerance, and insulin sensitivity, de Haan et al^.^[29]
2	CD151	0.856	0.864	Laminin was shown to regulate energy expenditure and insulin sensitivity [30]
3	GUCY2C	0.876	0.946	Silencing of the GUCY2C gene in mice disrupts satiation, resulting in hyperphagia and subsequent obesity and metabolic syndrome, Valentino et al. [31]
4	HCRTR2	0.85	0.851	Effects of orexins on energy metabolism and adipose tissue development [32]
5	ITGA7	0.872	0.940	ITGA7 is suggested to be responsible for laminin-dependent signaling in differentiating preadipocytes. Adipose tissue laminins regulate energy expenditure and insulin sensitivity (Morandi et al. [33] and Goddi et al. [30])
6	MCAM	0.860	0.879	MCAM is the laminin alpha 4 receptor that is related to obesity [34], adipose tissue expansion, and weight gain [35,36]
7	PDE1B	0.879	0.905	
8	PTPRS	0.852	0.860	The study identified several CpG methylation sites and specifically CpG sites located in PTPRS and PER3 genes differentially methylated between obese and non-obese children, suggesting that the epigenetic regulation of these CpGs might be involved in the development of childhood obesity (Samblas et al. [37])
9	TNFRSF1B	0.906	0.939	The M196R (676 T3G) variant in exon 6 of TNFRSF1B is associated with hyperandrogenism and PCOS, further suggesting a role for inflammatory cytokines in the pathogenesis of these disorders (Peral et al. [38]) GeneCards [39] Summary for the TNFRSF1B gene: this gene participates in “Cytokine Signaling in Immune System KEGG pathways”
10	ITGB3	0.924	0.932	
11	BDKRB2	0.933	0.931	
12	F2RL3	0.913	0.930	
13	OSMR	0.926	0.927	Suggest that adipocyte OSMR signaling is involved in the regulation of adipose tissue homeostasis and that in obesity, OSMR ablation may exacerbate insulin resistance by promoting adipose tissue inflammation (Carrie et al. [40])
14	F11R	0.923	0.925	
15	JMJD6	0.933	0.913	Demethylase JMJD6 as a new regulator of interferon signaling: Effects of HCV and Ethanol Metabolism (Murali et al. [41])
16	TACR1	0.875	0.912	
17	CD93	0.917	0.907	GeneCards [39] related pathway: “Immune response Lectin induced complement pathway” and Lee et al. [42]
18	IL18RAP	0.932	0.891	Genecards [39] related pathway: “Cytokine Signaling in Immune System” and Aqrawi et al. [43]
19	SELL	0.932	0.883	GeneCards [39]: “The gene product is required for binding and subsequent rolling of leucocytes on endothelial cells, facilitating their migration into secondary lymphoid organs and inflammation sites”
20	CSF3R	0.934	0.882	Showed increased expression of the CSF3R gene, which plays an essential role in the host immune response or the host defense against several pathogens or oxidative stress. GeneCards [39] related pathway: “Cytokine Signaling in Immune System” and Naruse et al. [44]
21	CD79A	0.940	0.878	CD79A is a lymphocyte receptor that is exclusively regulated in CD-MAT, exhibiting a different pattern of immune cell activation compared to the ileal mucosa in CD patients (Da Silva et al. [45])
22	FLT1	0.924	0.873	
23	FPR2	0.918	0.870	The endogenous anti-inflammatory role of murine Fpr2 was recently demonstrated in Fpr2–/–mice (Hellmann et al. [46])

## Discussion

Our approach predicts new metabolic and inflammatory functions of receptors in subcutaneous and visceral adipose tissues using a feature space of pathway enrichment analysis scores and co-expression modules’ eigengene correlations. For the analysis, we employed the XGBoost, linear SVM, and k-NN classifiers. We tested our technique on subcutaneous and visceral adipose RNA-seq data derived from the GTEx project [19]. Our approach detected metabolic and inflammatory receptors successfully in both tissues with an accuracy of 0.89 and 0.8 for adipose subcutaneous and visceral, respectively. The XGBoost model outperformed the linear SVM and k-NN approaches and was further used for feature analysis and predicting new functions of unlabeled receptors.

Interestingly, several receptors were misclassified by the classifiers, i.e., classified by the classification model differently than the original known label, in both tested adipose tissues. One misclassified gene is the EPOR gene, which was initially labeled by us as “inflammatory” since it is a member of the KEGG “cytokine-cytokine receptor interaction” pathway. The EPOR gene was misclassified by the XGB model as a “metabolic” receptor for both subcutaneous and visceral adipose, as noted in Table 3.

We found experimental support for model’s prediction that the cytokine receptor EPOR has a metabolic role in adipose and was shown to affect metabolic and glucose homeostasis in white adipose tissue [47–50]. Another example is the TNFRSF21 cytokine receptor derived from KEGG “cytokine-cytokine receptor interaction” pathway and which was misclassified as a “metabolic” receptor in both adipose tissues and is found to be related to the “regulation of lipid metabolic process” in GO. An additional example is the TNFRSF25 receptor, a member of the tumor necrosis factor receptor superfamily 25, which mediates apoptotic signaling and differentiation [51]. Its only known ligand is the TNF-like protein 1A (TL1A) [52], which is a pro-inflammatory cytokine. Interestingly and supporting our predictions, the TL1A ligand was shown to play an important role in regulating adipose tissue mass [53]. This evidence that supports our predictions for metabolic functions in both adipose tissues of known inflammatory receptors demonstrates the necessity of understanding the tissue-specific function of each receptor and the ability of inflammatory receptors to change their functions and effects within or across tissues [54–56]. Chen et al. [54] reviewed the roles of different pro-inflammatory cytokines in lipid metabolism of metabolic diseases including cancer and presented a list of these metabolic cytokines. Shi et al.[55] investigated the potential of receptors to modify their activity across tissues and discovered clear evidence that different types of cytokines contribute significantly to the development of abnormal glucose and lipid metabolism. Tumor necrosis factor (TNF) is one example of a pro-inflammatory cytokine and the first ’adipokine’ reported to be created by adipose tissue, regulated in obesity and related to obesity-related metabolic disease. TNF became characterized as an adipokine following the accidental discovery of its enhanced synthesis in adipose tissue in obesity, which led to an understanding of the inflammatory nature of obesity and accompanying metabolic disorders [56]. Here, we focused on the changing roles of receptors across tissues and used the model’s prediction probabilities (using classification probability cutoff > 0.85) to predict the main role of each receptor within a tissue.

We predicted eight new unlabeled receptors to be “metabolic” in both adipose tissues, as listed in Table 3 above. We found literature-based experimental support that many of them or their ligands exhibit metabolic functions (see Table 3 column 5). For example, the ABCA1 receptor was verified as regulating adipose tissue lipid content, glucose tolerance, and insulin sensitivity by de Hann et al. [29]. Another example is the GUCY2C receptor that was shown to disrupt satiation, resulting in hyperphagia and subsequent obesity and metabolic syndrome when silenced in mice by Valentino et al.[31]. MCAM is the laminin alpha 4 receptor that was related to obesity [34], adipose tissue expansion, and weight gain [35,36]. CD151 is a laminin receptor. Laminin was shown to regulate energy expenditure and insulin sensitivity [30]. HCRCR2, the hypocretin receptor type 2, is a receptor of the hypocretin (Orexin) ligand. Orexins/Hypocretins were shown to affect energy metabolism and adipose tissue development [32]. We predicted that 15 unlabeled receptors were “inflammatory” in both tissues, as listed in Table 3. We found experimental support that eight exhibit inflammatory-related roles. For example, the OSMR receptor in adipocytes was suggested to be involved in adipose tissue inflammation (Carrie et al. [40]). Demethylase JMJD6 is suggested to be a new regulator of interferon signaling [41]. These new predictions can now be further tested experimentally.

The feature analysis that we conducted verified that the biological pathways mostly discriminating scores used for the predictions were relevant to the predicted receptor types within each examined tissue. For example, the XGBoost model detected that 4 out of the 7 most predictive pathways of metabolic receptors in adipose subcutaneous were metabolic pathways, classified as “Metabolism”. Inflammatory-related pathways, classified as “Immune systems/Disease”, were highly significant for classifying the inflammatory receptors (see Fig 3). Furthermore, we demonstrated (see Figs 3 and 4 and S5 and S6 Figs in S1 File) how the most significant pathways affect the classification.

We note the limitation in that the GTEx data include bulk gene expression data comprising the gene expression of the cell types included in each tissue. Thus, the co-expression networks we detected here represent a combination of the expression of genes derived from the cellular composition of each tissue type. For example, part of the inflammatory receptor classification may be related to a signal that stems from inflammatory receptors located on immune cells in the adipose tissues. Another limitation is that the known labeled receptors list is relatively small (~50 receptors per class) which may result in a model trained on a subset of all possible features (biological pathways). Our models were trained with the biological pathways related to the labeled receptors. Thus, the model can infer the roles of unlabeled receptors that are enriched with biological pathways that participated in the training process. Finally, as noted before, receptors may exhibit multiple roles across tissues [55]. In this work we aimed to define the main changing roles across tissues. We used high cutoffs for predictors’ probabilities to infer these main roles.

In future work, we plan to extend this work with additional classes of receptors and additional tissues. We note that the extension of the proposed methodology to other tissues is challenging and may require the usage of more general features and the creation and validation of new tissue-specific labeled lists of “metabolic”, “inflammatory”, and “other” classes of receptors, which is poorly known for most of these other tissues.

In summary, our approach is successful in predicting the tissue-specific metabolic and inflammatory roles of receptors for adipose tissues. Our approach can save time by pinpointing the biological scientist and drug developer on disease-related potential receptors that should be further investigated and experimentally validated. In addition, our approach enabled us to draw a comprehensive and simultaneous view of the changing functions of receptors across tissues and throughout the body.

## Methods

### Ethics statement

The GTEx [19] v8 data was downloaded from https://gtexportal.org/home/datasets. The GTEx project follows all ethical, legal and social issues as detailed in the GTEx original publication [19]. For deceased donors to participate in GTEx, next of kin permission was obtained in writing or verbally, typically as part of an amendment to an existing authorization form for a donation of tissue or organ. It included statements common to consent forms, such as the intention to perform genetic analyses, establish cell lines, and share data with the scientific community. Surgical living donors are only allowed to participate after obtaining written informed consent.

### Data preprocessing

The GTEx database [19] (v8) was used to download RNA-seq data from 54 human tissues and 17,382 RNA-seq samples from 948 donors. The transcripts per million (TPM) values were then log2-transformed. We used the data of visceral adipose and subcutaneous adipose. To include reasonably healthy donors, samples with death circumstance #4 (slow death after a long illness) were removed. All genes within each tissue were quantile normalized, and outlier samples were filtered (to remove background and sample effects). Genes with zero variance or missing samples were omitted from the analysis. Genes that had at least 0.1 TPM in at least 80% of the samples were kept. Outliers were removed using the isolation forest model [57,58].

### Outlier removal

For outlier removal, we used an isolation forest-based approach [57] that uses an ensemble of machine learning trees to isolate anomalous points in the dataset (see the explanation in S7 Fig in S1 File).

### Confounding factors adjustments

Somekh et al. [59] showed that correcting for known confounding factors, e.g., by using linear regression based-adjustment of the heterogenous GTEx data, outperform other methods in preserving the biological signal–which is relevant here. Thus, we used linear regression models to adjust for the known confounding factors: experimental batch, ischemic time (elapsed time between actual death and sample extraction), gender, age, and death circumstances.

The age factor covered ages 20–80 and is partitioned into 10-year intervals (embedded in the GTEx dataset). The samples’ circumstances of death type classification (DTHHRDY) is based on a four-point Hardy Scale: 0 = cases on mechanical ventilator prior death, 1 = non-ventilation fast deaths due to accident, blunt force, trauma, or suicide of healthy individuals, 2 = non-ventilation fast deaths of natural causes of healthy individuals, 3 = intermediate death after a terminal phase of 1 to 24 hours, and 4 = slow death after a long illness. As the focus of our research was on relatively healthy individuals at the time of death, we excluded samples with a DTHHRDY value of 4, non-healthy individuals with a long-term illness that also includes a small number of samples.

We performed linear regression to correct for the known confounding factors (age, sex, batch, ischemic time, and death circumstances) as follows:

$${Residual}_{i}^{j}=Ex{p_{i}}^{j}-\sum_{n=1}^{N}{Coef}_{i,n}\times Confounde{r_{n}}^{j}$$ (1)

${Exp}_{i}^{j}$ is the expression level of gene *i* in sample *j*, *Coef*_*i*,*n*_ is the confounding coefficients of the *n*^th^ confounder in the regression model of gene *i*, and *Confounder*_*n*_^*j*^ is the value of confounder *n* in sample *j*. The residuals were used as the input data for the co-expression module detection.

### Co-expression module detection

The most common algorithm for co-expression network analysis is WGCNA implemented in the WGCNA R package [8]. Using correlation coefficient *cor*(*i*,*j*), the method created a similarity co-expression matrix for all genes (we used the biweight midcorrelation measure that accounts for outliers, by assigning larger weights to values closer to medians). The soft thresholding power *β* is used to mimic a scale-free network and to increase the co-expression similarity. The resulting co-expression network is presented by an adjacency matrix.

$$a_{ij}={\left(0.5*(1+cor(1,j))\right)}^{\beta }$$ (2)

where *a*_*ij*_ is the resulting adjacency that measures the strength of the connections. We determined the soft-thresholding power *β* for network construction parameter as 14, by using the criterion of approximating the network’s scale-free topology as suggested in the algorithm [8] (it can be seen in S8A Fig in S1 File that an “elbow” form corresponds to a *β* = 14). The dissimilarity TOM is then computed from a topological overlap matrix (TOM) [8]. The TOM calculated the topological similarity between each pair of neighbors in the network, i.e., it compared the neighbors of each pair of nodes. Finally, the dissimilarity TOM was utilized to create a tree (dendrogram) using hierarchical clustering. Clusters (modules) are obtained from the tree using dynamic tree cutting. The resulting modules featured tightly coupled genes, allowing co-expression networks, also known as modules, to be constructed for each tissue. The “signed” parameter was employed to characterize the positively/negatively correlated genes in distinct modules, meaning that the co-expressed modules include only positively correlated genes. Eigengenes are the weighted average of each module’s expression profile and are defined as the first principal component of the expression matrix of the genes in each module. The module membership (kME) measures can be defined (also known as eigengene-based connectivity) by calculating the correlation between each gene in the module and the module’s eigengene. The eigengenes can be further utilized to merge clusters and screen for prospective gene targets using the dendrogram cut height as a module merging parameter. We set the module merging parameter to 0.25, corresponding to a correlation of 0.75 between the module’s eigengenes. We tested several values for this parameter to find the best one for merging vis-à-vis the model’s performances. Using this method, the essential driver genes, the kMEs, in each module were identified.

### KEGG enrichment analysis of modules

KEGG is a database resource comprising cellular biological pathways. We used the R tool ‘clusterProfiler^’^ [60] on all 548 KEGG pathways to generate pathway enrichment analysis of the modules based on the hypergeometric test. The significance of the pathways in each co-expression module was represented by the log-transformed adjusted p-values (adjusted for multiple corrections using the BH (Benjamini and Hochberg) method [61]) and were used as the features for the machine learning classification models.

### Machine learning models

The machine learning methods that we used in this work are:

#### K-Nearest Neighbors (k-NN)

The k-NN algorithm is a distance-based learning algorithm used for classification [62,63]. The algorithm takes, as input, the k closest labeled examples in the feature space. The most common distance measure is the Euclidean distance. A data point is classified by a plurality vote of its neighbors, with the data point being assigned to the most common class among its k nearest neighbors (k is a positive integer, typically small). The k-NN’s performance is very sensitive to the choice of k and an optimal k can be selected by various heuristic techniques [64]. A common way of choosing the empirically optimal k is by testing the error rate under a set of possible k values.

#### Support Vector Machine (SVM)

SVM is a classification method that has been proved to work in a range of situations [18]. Based on their properties of belonging to a class, a linear SVM generates a hyperplane that separates positive and negative samples. SVM can be used with different kernels such as linear and Gaussian kernels. The linear kernel will create a straight line as the decision boundary, making the data linearly separable, while the Gaussian (RBF) kernel will project the data into a Gaussian distribution. The SVM linear kernel is best used to avoid overfitting when dealing with small sample sizes. For the SVM computations, we used the Python scikit-learn SVM package.

#### XGBoost

The XGBoost approach [65] is a boosted decision tree approach that is based on Friedman’s gradient boosting [66,67] and incorporates extra enhancements that improve the results’ performance and accuracy. While the trees in the original gradient boosting model are produced in sequence, XGBoost builds them in parallel, similar to the random forest approach, where each tree attempts to compensate for the areas where the preceding tree was less accurate. Regularization terms are also used in this method for managing the variance of the fit and the flexibility of the learning task, resulting in models that generalize better to unknown data as opposed to other machine learning models. The XGBoost [65] technique works well on small samples and a large number of features. Additionally, tree boosting machines have explainability capabilities, which can aid in evaluating the model’s correctness by examining the relevance of the most important features to the phenotype. In addition, the XGBoost model enables handling missing values uniquely. For example, during splitting, XGBoost will allocate all missing data to the node which will mostly improve the model’s prediction performance. In our scenario this ability is very useful since there are features with missing values, i.e., not every receptor (module) has an enrichment score for each biological pathway.

We used the XGBoost python library [68] on our case of a small sample with a large number of features. In addition to employing XGBoost to predict the class of each receptor, we used the predict_proba() function to get the probability of each receptor to belong to each class. These probabilities are calculated based on the number of votes for each class divided by the number of trees, e.g., the number of votes each receptor received for each class by each tree divided by the n_estimators (number of trees in the model).

### Positive unlabeled (PU) SVM bagging

Supervised learning necessitates the definition (labeling) of positive and negative training instances. Obtaining negative examples is more expensive than obtaining positive examples in most fields, and it is occasionally impossible. Unlabeled examples are those in which we do not know whether they are positive or negative. For example, if a receptor has been proven to be metabolic in an experiment, we label/annotate it as a positive metabolic; however, we are unsure and lack the expertise to annotate the non-metabolic receptors, which are the unlabeled receptors.

PU learning algorithms [11] comprise a group of algorithms meant to learn from a small number of positive instances and a large number of unlabeled examples, in the absence of negative examples. The majority of these algorithms rely on traditional supervised classification methods such the SVM classifier. Kiliç and Tan [11], for example, examined eight PU learning techniques for detecting protein–protein interaction (PPI) networks from gene expression data using just positive prior knowledge of known protein–protein interactions. The PU bagging SVM algorithm [69] is an effective algorithm for this goal. In each iteration of the algorithm, a random subset of the unlabeled set is specified as containing the negative examples (under the assumption that most of the unlabeled examples are negative), and a classifier is trained using this negative subset and the known positive examples. Finally, by executing multiple iterations, the negative and positive rates for each example are calculated using the combined results of these numerous classifiers. The method, in other words, (1) builds a training set by mixing all positive data points with a random sample from the unlabeled points, by replacement; (2) it creates an SVM classifier from this “bootstrap” sample, treating positive and unlabeled data points as positives and negatives, respectively; (3) it then applies the generated SVM classifier to the rest of the unlabeled data points not included in the trained random sample–hereafter referred to as OOB (“out of the bag”) points–for prediction and records their scores; (4) the three steps above are repeated multiple times, and finally, the average of the OOB scores each point has received are assigned to it, e.g., the rate at which all receptor predictions are classified as non-metabolic or metabolic. After more than 100 iterations of the PU bagging algorithm, there was little improvement in simulated and real data [11,69]. Even when the number of known positives is low, the bagging SVM approach beats state-of-the-art methods for PU learning [11,69] and successfully discriminates between unlabeled positive and negative samples.

We utilized the PU SVM bagging method, dedicated for binary classification, to define the third (“other”) class representing the non-metabolic and non-inflammatory receptors. To generate a list of negative receptors that are non-metabolic and non-inflammatory, we used the “metabolic” labeled receptors as the positive labeled group against all other receptors designated as the unlabeled group. The known inflammatory receptors (derived from the KEGG cytokine–cytokine receptor interaction list) were excluded from the unlabeled group. Using the PU SVM algorithm, we retained the top 50 “negative” receptors, i.e., the non-metabolic and non-inflammatory receptors, from this analysis to represent the “other” group. We retained 50 top "negative" receptors to maintain a balanced training set of ~50 labeled metabolic and inflammatory receptors.

### Cross-validation

Cross-validation [70] is a technique used when the annotated data is limited. This method splits the annotated dataset into a training set and a test set and evaluates the performance of a prediction model on data points that are not used to train the model. A popular method of cross-validation is sub-sampling (k-fold cross-validation). In k-fold cross-validation, as the name suggests, the dataset is randomly divided into k number of non-overlapping sets. During each iteration, one set is used as a test dataset and the rest are used for training the model. The test dataset is predicted by the trained model. This iteration is repeated k times, each time with different training and test groups, and generates k different classification models. The performance statistics are calculated by summing in each distinct test group the true positives, true negatives, false positives, and false negatives.

### Evaluation matrices

The performance of the classifiers was measured by examining how well the classifier identified the positive and negative or the multiclass examples in the test sets. For binary classification, each sample in the test set can be categorized as true positive (TP), true negative (TN), false positive (FP), or false negative (FN). We used accuracy, recall, precision, and F1 to evaluate the performance of the cross-validation analysis. The mathematical equations to calculate these parameters are as follows:

$$Accuracy=\frac{TP+TN}{TP+FP+TN+FN}$$

$$Recall=\frac{TP}{TP+FN}$$

$$Precision=\frac{TP}{TP+FP}$$

$$F1=\frac{TP}{TP+\frac{1}{2}(FP+FN)}$$

The overall correctly predicted examples were calculated by using accuracy. We calculated the average accuracy, precision, recall, and F1 per class. For example, overall accuracy for the three-class classifier was calculated as follows:

$$Average\;Accuracy=\frac{\sum_{i=1}^{k=3}\frac{{TP}_{i}+{TN}_{i}}{{TP}_{i}+{FP}_{i}+{TN}_{i}+{FN}_{i}}}{k}$$

### Feature analysis

SHAP–SHapley Additive exPlanations [26], Lundberg’s approach for explaining boosted trees, was used for features analysis. SHAP is a fast, accurate technique that can explain the results of any machine learning model, including tree ensemble methods. SHAP generates values for each feature, which are the average marginal contribution of the feature across all permutations, indicating how much each feature contributes to pushing the model output from the base value (the average model output across the training dataset we provided) to the model output. In the model’s trees, features enter the machine learning model sequentially and repeatedly. The algorithm assesses each feature equally at each level of tree growth to determine which feature contributes the most. Hundreds of thousands of trees are planted. Different combinations of features may be offered. As a result, each feature’s marginal contribution may be computed.

### Methodology and experimental design

We preprocessed the data and accounted for batch effects as described in the Methods section.

### Receptor labeling

We retained a list of ~700 known receptors from Ramilowski et al [28]. We labeled the genes that correspond to known receptors into “metabolic”, “inflammatory”, or “other” receptors. A list of 52 positive labeled metabolic receptors for both tissues was taken from Somekh et al [23]. Somekh et al. [23] labeled metabolic receptors in subcutaneous adipose based on a semi-supervised approach, using the SVM PU bagging algorithm, and a literature verification using published experiments. Inflammatory receptors were derived from the KEGG cytokine–cytokine receptor interaction list downloaded from the KEGG database. We retained only genes that were included in our known receptor list and only receptors that were not labeled as “metabolic” by Somekh et al [23]. Additionally, we filtered out inflammatory receptors that are related to metabolic/growth regulation processes according to the GO database. For filtering the KEGG’s cytokine receptors, we used all the GO molecular functions and processes that were marked by Somekh et al. [23] (the full list of processes is available in S3 Table in S1 File). The “other” group receptors were labeled using semi-supervised learning by running the SVM PU bgagging algorithm where the top 50 “negative” (non-metabolic and non- inflammatory) receptors were used.

### Co-expression and enrichment analysis

We generated co-expression networks for both tissues (subcutaneous adipose and visceral adipose) and annotated the modules using KEGG pathway enrichment analysis. Pathway enrichment analysis was done for each modules separately. Those pathways that did not contain any genes from the module had null values as their enrichment score. This way the number of non-null features (pathways) varied between the modules. All KEGG enrichment scores were log2 transformed to normalize the skewness of the scores. These scores were used as the machine learning models’ features. We learned about the function of the receptor from the known functions of the genes included in its composing co-expression network. For each tested receptor, the enrichment scores of its composing co-expression network were used.

### Classifier construction and validation

We employed SVM, k-NN, and XGBoost models to solve a three-class receptor classification problem for the “metabolic”, “inflammatory” and “other” receptor lists. We set up the models for each of the two adipose tissues to use the tissues’ module enrichment scores and the correlation between the modules’ eigengenes as features, together with the correlation of each receptor to each module. For cross-validation, we utilized a non-shuffled scikit-learn implementation of Stratified K-Fold Cross-Validation [71]. This cross-validation is a K-Fold variant that yields stratified folds. The folds are created by keeping track of the percentage of samples in each class. We picked a 10:90 split, which means that 90% of the data is used for training and 10% for validation each time.

We fine-tuned each model’s hyperparameters to find the best estimator according to the cross-validation. We tested the following parameters for the XGBoost model: number of estimators (trees) 100 or 300, max depth (how deeply each tree is allowed to grow during any boosting round) of 3 or 5, learning rates (step size shrinkage used to prevent overfitting) of 0.01, 0.03, or 0.09, a subsample (percentage of samples used per tree) of 0.9 or 1.0, colsample_bytree (percentage of features used per tree) of 0.3, 0.5 or 0.9, and gamma (regularization parameter that controls whether a given node will split) of 0, 1 or 5. The best estimator model was then used for generating the predictions.

The experiment contained a 10-fold cross-validation prediction, with the results of the ten executions being saved. The results of the ten executions, as well as the features’ importance, were averaged. We tracked the average and standard deviation of each experiment’s accuracy, precision, recall, and F1 score for each prediction.

### Predictions and feature analysis

We executed feature analysis using the SHAP [26] for explaining boosted trees to find the most predictive features (KEGG biological pathways and WGCNA module correlations). For prediction purposes, we employed our best cross-validated model to categorize the unlabeled receptors. The study comprised 323 unlabeled receptors that we classified. These were not part of the training–test process and were included in modules in subcutaneous or visceral adipose.

## Supporting information

S1 File Supplementary material to the manuscript. (DOCX)
