# Supplementary material for: A methodology for classifying tissue-specific metabolic and inflammatory receptor functions applied to subcutaneous and visceral adipose
Source: PLoS One. 2022 Oct 25;17(10):e0276699. doi: 10.1371/journal.pone.0276699 (PMC9595531; doi:10.1371/journal.pone.0276699)
Supplement: S1 File — (DOCX) [file pone.0276699.s001.docx]

# **Supplementary Information**

**A methodology for classifying tissue-specific metabolic and inflammatory receptor functions applied to subcutaneous and visceral adipose**

**Gur Arye Yehuda**^1^ **and Judith Somekh**^1,*^

^1^University of Haifa, Information Systems, Haifa, 3498838, Israel

^*^judith_somekh@is.haifa.ac.il

## **S1 Table – New predicted metabolic receptors**

Lists of the highest (probability >0.85) predicted metabolic receptors for both adipose tissues.

Adipose Subcutaneous

| **Gene Symbol** | **Prediction Probability** |
| --- | --- |
| FZD9 | 0.910 |
| P2RY12 | 0.898 |
| ITGAE | 0.896 |
| OPRK1 | 0.888 |
| GPR37 | 0.882 |
| VANGL1 | 0.880 |
| PDE1B | 0.880 |
| SDC1 | 0.878 |
| GUCY2C | 0.877 |
| ABCA1 | 0.876 |
| ITGA7 | 0.873 |
| GPC1 | 0.871 |
| TNFRSF21 | 0.869 |
| FZD7 | 0.868 |
| PDE1C | 0.867 |
| NTSR2 | 0.866 |
| TRPC3 | 0.865 |
| MCAM | 0.860 |
| CRLF1 | 0.860 |
| HTR2A | 0.857 |
| HCRTR1 | 0.857 |
| CD151 | 0.856 |
| PROKR1 | 0.853 |
| PTPRS | 0.853 |

Adipose Visceral

| **Gene Symbol** | **Prediction Probability** |
| --- | --- |
| GUCY2C | 0.947 |
| ITGA7 | 0.940 |
| PLXNB3 | 0.930 |
| OPRD1 | 0.924 |
| RTN4RL1 | 0.922 |
| IL17RC | 0.918 |
| LHCGR | 0.911 |
| ADRB3 | 0.910 |
| ABCA1 | 0.909 |
| PDE1B | 0.905 |
| LINGO1 | 0.904 |
| ITGA8 | 0.903 |
| ADRA2A | 0.901 |
| CLDN2 | 0.894 |
| MCAM | 0.880 |
| NTRK3 | 0.877 |
| EDNRA | 0.876 |
| TBXA2R | 0.870 |
| CD151 | 0.864 |
| PTPRS | 0.860 |
| HCRTR2 | 0.852 |
| LGR4 | 0.851 |

## **S2 Table – New predicted inflammatory receptors**

Lists of the highest (probability >0.85) predicted inflammatory receptors for both adipose tissues.

Adipose Subcutaneous

| **Gene Symbol** | **Prediction Probability** |
| --- | --- |
| CD79A | 0.940 |
| CXCR3 | 0.940 |
| IL7R | 0.938 |
| CXCR4 | 0.938 |
| KLRC2 | 0.937 |
| KLRC1 | 0.936 |
| CXCR2 | 0.934 |
| KCNA3 | 0.934 |
| CSF3R | 0.934 |
| BDKRB2 | 0.933 |
| JMJD6 | 0.933 |
| NRP2 | 0.933 |
| SELL | 0.932 |
| IL18RAP | 0.932 |
| FCGR1A | 0.932 |
| CD5 | 0.931 |
| KLRD1 | 0.930 |
| BDKRB1 | 0.928 |
| CD3G | 0.927 |
| OSMR | 0.926 |
| KIR2DL1 | 0.926 |
| ITGB7 | 0.925 |
| ITGB3 | 0.924 |
| FLT1 | 0.924 |
| S1PR5 | 0.924 |
| KCNJ15 | 0.923 |
| F11R | 0.923 |
| KIR3DL2 | 0.923 |
| CD247 | 0.923 |
| ITGAL | 0.922 |
| SIRPG | 0.921 |
| TRAF2 | 0.920 |
| KLRK1 | 0.919 |
| CCRL2 | 0.919 |
| FPR2 | 0.918 |
| SLC37A1 | 0.918 |
| ITGA4 | 0.917 |
| CD93 | 0.917 |
| CD3D | 0.917 |
| NCR3 | 0.916 |
| CLEC2B | 0.914 |
| F2RL3 | 0.913 |
| SELE | 0.912 |
| SLC18A2 | 0.911 |
| TNFRSF1B | 0.906 |
| FPR1 | 0.905 |
| PDGFRA | 0.905 |
| LPAR1 | 0.905 |
| NRP1 | 0.901 |
| MRGPRX2 | 0.901 |
| KIT | 0.900 |
| AMHR2 | 0.899 |
| IL17RB | 0.895 |
| AVPR1A | 0.891 |
| ATP6AP2 | 0.891 |
| CD40 | 0.889 |
| APLNR | 0.888 |
| ACKR2 | 0.888 |
| ITGA1 | 0.888 |
| S1PR1 | 0.886 |
| TMEM222 | 0.886 |
| LRP4 | 0.881 |
| IFITM1 | 0.880 |
| ROR1 | 0.880 |
| SDC2 | 0.879 |
| ACVR1 | 0.878 |
| CD74 | 0.878 |
| CD9 | 0.878 |
| EPHA5 | 0.877 |
| TACR1 | 0.875 |
| HLA-F | 0.873 |
| AQP1 | 0.872 |
| HFE2 | 0.872 |
| NTRK2 | 0.871 |
| ITGA11 | 0.871 |
| FZD1 | 0.870 |
| FCER1A | 0.870 |
| TGFBR3 | 0.869 |
| PTPRZ1 | 0.869 |
| GRIN2D | 0.869 |
| NEO1 | 0.868 |
| MAG | 0.867 |
| AXL | 0.867 |
| MYLK2 | 0.865 |
| ROBO2 | 0.863 |
| ERBB3 | 0.863 |
| KCNJ10 | 0.862 |
| SORCS3 | 0.859 |
| NRXN1 | 0.859 |
| CDH2 | 0.857 |
| HMMR | 0.857 |
| ACKR4 | 0.857 |
| THBD | 0.856 |
| ITGB8 | 0.855 |
| GPR37L1 | 0.854 |
| CDH19 | 0.853 |

Adipose Visceral

| **Gene Symbol** | **Prediction Probability** |
| --- | --- |
| FZD10 | 0.942 |
| TNFRSF9 | 0.941 |
| IL1RL2 | 0.941 |
| PVR | 0.941 |
| TNFRSF1B | 0.939 |
| CD44 | 0.935 |
| S1PR3 | 0.933 |
| ITGB3 | 0.932 |
| BDKRB2 | 0.931 |
| F2RL3 | 0.930 |
| BMPR2 | 0.928 |
| OSMR | 0.927 |
| IL6R | 0.927 |
| GPR75 | 0.926 |
| F11R | 0.925 |
| TNFRSF17 | 0.923 |
| ASGR1 | 0.921 |
| TGFBR1 | 0.917 |
| JMJD6 | 0.913 |
| ASGR2 | 0.913 |
| TACR1 | 0.912 |
| TNFRSF12A | 0.911 |
| CD93 | 0.907 |
| ITGA10 | 0.902 |
| MC1R | 0.901 |
| IL18RAP | 0.891 |
| ITGA5 | 0.889 |
| PLXNA2 | 0.887 |
| SORL1 | 0.887 |
| SIRPB1 | 0.884 |
| SELL | 0.883 |
| CSF3R | 0.882 |
| CD79A | 0.878 |
| BOC | 0.874 |
| IL13RA2 | 0.874 |
| ALOX5 | 0.873 |
| FLT1 | 0.873 |
| MRC2 | 0.872 |
| CD19 | 0.872 |
| ORAI2 | 0.871 |
| FPR2 | 0.870 |
| TSPAN1 | 0.869 |
| C5AR2 | 0.869 |
| SMO | 0.859 |
| FZD7 | 0.856 |
| HRH2 | 0.853 |

## **S3 Table – Gene Ontology metabolic terms**

The list of GO (Gene Ontology) terms we used to filter inflammatory receptors that exhibit metabolic functions. The list is taken from Somekh et al^10^, supplemental table S2.

| Ontology Term Identifier | Ontology Term Name | Ontology Term Namespace |
| --- | --- | --- |
| GO:0007155 | cell adhesion | biological_process |
| GO:0030054 | cell junction | cellular_component |
| GO:0007275 | multicellular organism development | biological_process |
| GO:0008285 | negative regulation of cell population proliferation | biological_process |
| GO:0030335 | positive regulation of cell migration | biological_process |
| GO:2000810 | regulation of bicellular tight junction assembly | biological_process |
| GO:0071329 | cellular response to sucrose stimulus | biological_process |
| GO:0006897 | endocytosis | biological_process |
| GO:0034446 | substrate adhesion-dependent cell spreading | biological_process |
| GO:0071222 | cellular response to lipopolysaccharide | biological_process |
| GO:0001558 | regulation of cell growth | biological_process |
| GO:0060070 | canonical Wnt signaling pathway | biological_process |
| GO:0001938 | positive regulation of endothelial cell proliferation | biological_process |
| GO:0045600 | positive regulation of fat cell differentiation | biological_process |
| GO:0007584 | response to nutrient | biological_process |
| GO:0019222 | regulation of metabolic process | biological_process |
| GO:0050796 | regulation of insulin secretion | biological_process |
| GO:0008284 | positive regulation of cell population proliferation | biological_process |
| GO:0050731 | positive regulation of peptidyl-tyrosine phosphorylation | biological_process |
| GO:0002021 | response to dietary excess | biological_process |
| GO:0009749 | response to glucose | biological_process |
| GO:0019838 | growth factor binding | molecular_function |
| GO:0019915 | lipid storage | biological_process |
| GO:0032868 | response to insulin | biological_process |
| GO:0046676 | negative regulation of insulin secretion | biological_process |
| GO:0071363 | cellular response to growth factor stimulus | biological_process |
| GO:0030308 | negative regulation of cell growth | biological_process |
| GO:0016042 | lipid catabolic process | biological_process |
| GO:0055089 | fatty acid homeostasis | biological_process |
| GO:0070328 | triglyceride homeostasis | biological_process |
| GO:0008283 | cell population proliferation | biological_process |
| GO:0030206 | chondroitin sulfate biosynthetic process | biological_process |
| GO:0030208 | dermatan sulfate biosynthetic process | biological_process |
| GO:0048008 | platelet-derived growth factor receptor signaling pathway | biological_process |
| GO:0048771 | tissue remodeling | biological_process |
| GO:0005104 | fibroblast growth factor receptor binding | molecular_function |
| GO:0005975 | carbohydrate metabolic process | biological_process |
| GO:0008543 | fibroblast growth factor receptor signaling pathway | biological_process |
| GO:0017134 | fibroblast growth factor binding | molecular_function |
| GO:0090080 | positive regulation of MAPKKK cascade by fibroblast growth factor receptor signaling pathway | biological_process |
| GO:0006091 | generation of precursor metabolites and energy | biological_process |
| GO:0042417 | dopamine metabolic process | biological_process |
| GO:0008203 | cholesterol metabolic process | biological_process |
| GO:0015485 | cholesterol binding | molecular_function |
| GO:0042632 | cholesterol homeostasis | biological_process |
| GO:0071404 | cellular response to low-density lipoprotein particle stimulus | biological_process |
| GO:0008289 | lipid binding | molecular_function |
| GO:0061178 | regulation of insulin secretion involved in cellular response to glucose stimulus | biological_process |
| GO:0005543 | phospholipid binding | molecular_function |
| GO:0010641 | positive regulation of platelet-derived growth factor receptor signaling pathway | biological_process |
| GO:0001523 | retinoid metabolic process | biological_process |
| GO:0002053 | positive regulation of mesenchymal cell proliferation | biological_process |
| GO:0010715 | regulation of extracellular matrix disassembly | biological_process |
| GO:0005518 | collagen binding | molecular_function |
| GO:0097009 | energy homeostasis | biological_process |
| GO:0016787 | hydrolase activity | molecular_function |
| GO:0004967 | glucagon receptor activity | molecular_function |
| GO:0016519 | gastric inhibitory peptide receptor activity | molecular_function |
| GO:0031018 | endocrine pancreas development | biological_process |
| GO:0032024 | positive regulation of insulin secretion | biological_process |
| GO:0070542 | response to fatty acid | biological_process |
| GO:0040037 | negative regulation of fibroblast growth factor receptor signaling pathway | biological_process |
| GO:0051004 | regulation of lipoprotein lipase activity | biological_process |
| GO:0051006 | positive regulation of lipoprotein lipase activity | biological_process |
| GO:0071813 | lipoprotein particle binding | molecular_function |
| GO:0001889 | liver development | biological_process |
| GO:0005041 | low-density lipoprotein particle receptor activity | molecular_function |
| GO:0030169 | low-density lipoprotein particle binding | molecular_function |
| GO:0030299 | intestinal cholesterol absorption | biological_process |
| GO:0034383 | low-density lipoprotein particle clearance | biological_process |
| GO:0070508 | cholesterol import | biological_process |
| GO:0042953 | lipoprotein transport | biological_process |
| GO:0045807 | positive regulation of endocytosis | biological_process |
| GO:0006007 | glucose catabolic process | biological_process |
| GO:0060612 | adipose tissue development | biological_process |
| GO:0001886 | endothelial cell morphogenesis | biological_process |
| GO:0010812 | negative regulation of cell-substrate adhesion | biological_process |
| GO:0048662 | negative regulation of smooth muscle cell proliferation | biological_process |
| GO:0042755 | eating behavior | biological_process |
| GO:0008035 | high-density lipoprotein particle binding | molecular_function |
| GO:0071223 | cellular response to lipoteichoic acid | biological_process |
| GO:0045599 | negative regulation of fat cell differentiation | biological_process |
| GO:0046323 | glucose import | biological_process |
| GO:0051005 | negative regulation of lipoprotein lipase activity | biological_process |
| GO:0001952 | regulation of cell-matrix adhesion | biological_process |
| GO:0030947 | regulation of vascular endothelial growth factor receptor signaling pathway | biological_process |
| GO:0010886 | positive regulation of cholesterol storage | biological_process |
| GO:0034197 | triglyceride transport | biological_process |
| GO:0044539 | long-chain fatty acid import into cell | biological_process |
| GO:0050909 | sensory perception of taste | biological_process |
| GO:0055096 | low-density lipoprotein particle mediated signaling | biological_process |
| GO:0070892 | lipoteichoic acid immune receptor activity | molecular_function |

## **S4 Table – Percentage of labeled receptors within representative WGCNA co-expression modules for Subcutaneous Adipose**

| Module # | Metabolic | Inflammatory | Other |
| --- | --- | --- | --- |
| 1 | 52% | 4% | 0% |
| 4 | 2% | 9% | 72% |
| 6 | 0% | 13% | 0% |
| 8 | 0% | 0% | 28% |
| 16 | 20% | 2% | 0% |
| 26 | 0% | 13% | 0% |

## **S5 Table – Percentage of labeled receptors within representative WGCNA co-expression modules for Visceral Adipose**

| Module # | Metabolic | Inflammatory | Other |
| --- | --- | --- | --- |
| 16 | 0% | 4% | 40% |
| 14 | 0% | 6% | 26% |
| 21 | 0% | 13% | 24% |
| 7 | 36% | 2% | 0% |
| 2 | 0% | 11% | 0% |
| 1 | 2% | 9% | 0% |

As shown, the majority of “metabolic” labeled receptors were clustered to modules 1 and 16 (52% and 20% respectively), Inflammatory receptors were clustered to modules 6, 26, and 4 (13%, 13%, and 9% respectively), and “other” receptors were clustered to modules 4 and 8 (72% and 28% respectively). Full labeled receptors distribution in all WGCNA generated modules are presented in supplemental table S7.

## **S6 Table – Receptors with different predictions for subcutaneous and visceral adipose. "Metabolic" prediction's probability is colored in green. "Inflammatory-related" prediction's probability is colored in pink.**

|  | **Receptor’s gene symbol** | **Class prob. Sub. adipose** | **Class prob. Vis. adipose** |
| --- | --- | --- | --- |
| 1 | EDNRA | 0.943 | 0.985 |
| 2 | APLNR | 0.994 | 0.959 |
| 3 | PTCH2 | 0.989 | 0.929 |
| 4 | BDKRB1 | 0.988 | 0.931 |
| 5 | NRP1 | 0.990 | 0.875 |
| 6 | PLXND1 | 0.970 | 0.887 |
| 7 | NRXN1 | 0.975 | 0.871 |
| 8 | MYLK2 | 0.980 | 0.877 |
| 9 | LRP6 | 0.938 | 0.910 |
| 10 | FZD10 | 0.977 | 0.984 |
| 11 | SMO | 0.954 | 0.969 |
| 12 | MC1R | 0.959 | 0.963 |
| 13 | MRC2 | 0.951 | 0.942 |
| 14 | GPC4 | 0.931 | 0.963 |
| 15 | BMPR2 | 0.899 | 0.985 |
| 16 | ITGA10 | 0.892 | 0.970 |
| 17 | HRH1 | 0.986 | 0.868 |
| 18 | SLC16A4 | 0.991 | 0.864 |
| 19 | EDA2R | 0.974 | 0.855 |

## **S7 Table – Labeled receptors distribution across WGCNA generated modules**

| **Subcutaneous Adipose** | | | |
| --- | --- | --- | --- |
| **Module** | **Type** | **# of receptors** | **% from group** |
| 0 | cytokine | 12 | 23% |
| 6 | cytokine | 7 | 13% |
| 26 | cytokine | 7 | 13% |
| 4 | cytokine | 5 | 10% |
| 30 | cytokine | 3 | 6% |
| 1 | cytokine | 2 | 4% |
| 55 | cytokine | 2 | 4% |
| 57 | cytokine | 2 | 4% |
| 59 | cytokine | 2 | 4% |
| 2 | cytokine | 1 | 2% |
| 9 | cytokine | 1 | 2% |
| 16 | cytokine | 1 | 2% |
| 18 | cytokine | 1 | 2% |
| 19 | cytokine | 1 | 2% |
| 21 | cytokine | 1 | 2% |
| 27 | cytokine | 1 | 2% |
| 38 | cytokine | 1 | 2% |
| 47 | cytokine | 1 | 2% |
| 58 | cytokine | 1 | 2% |
| 1 | metabolic | 26 | 52% |
| 16 | metabolic | 10 | 20% |
| 0 | metabolic | 6 | 12% |
| 2 | metabolic | 1 | 2% |
| 3 | metabolic | 1 | 2% |
| 4 | metabolic | 1 | 2% |
| 5 | metabolic | 1 | 2% |
| 27 | metabolic | 1 | 2% |
| 29 | metabolic | 1 | 2% |
| 34 | metabolic | 1 | 2% |
| 42 | metabolic | 1 | 2% |
| 4 | other | 36 | 72% |
| 8 | other | 14 | 28% |

| **Visceral Adipose** | | | |
| --- | --- | --- | --- |
| **Module** | **Type** | **# of receptors** | **% from group** |
| 21 | cytokine | 7 | 13% |
| 0 | cytokine | 6 | 11% |
| 2 | cytokine | 6 | 11% |
| 1 | cytokine | 5 | 9% |
| 18 | cytokine | 5 | 9% |
| 14 | cytokine | 3 | 6% |
| 22 | cytokine | 3 | 6% |
| 37 | cytokine | 3 | 6% |
| 4 | cytokine | 2 | 4% |
| 12 | cytokine | 2 | 4% |
| 16 | cytokine | 2 | 4% |
| 24 | cytokine | 2 | 4% |
| 3 | cytokine | 1 | 2% |
| 6 | cytokine | 1 | 2% |
| 7 | cytokine | 1 | 2% |
| 8 | cytokine | 1 | 2% |
| 15 | cytokine | 1 | 2% |
| 19 | cytokine | 1 | 2% |
| 31 | cytokine | 1 | 2% |
| 7 | metabolic | 18 | 36% |
| 0 | metabolic | 5 | 10% |
| 24 | metabolic | 5 | 10% |
| 6 | metabolic | 4 | 8% |
| 12 | metabolic | 3 | 6% |
| 26 | metabolic | 3 | 6% |
| 3 | metabolic | 2 | 4% |
| 4 | metabolic | 2 | 4% |
| 11 | metabolic | 2 | 4% |
| 1 | metabolic | 1 | 2% |
| 9 | metabolic | 1 | 2% |
| 13 | metabolic | 1 | 2% |
| 15 | metabolic | 1 | 2% |
| 18 | metabolic | 1 | 2% |
| 31 | metabolic | 1 | 2% |
| 16 | other | 20 | 40% |
| 14 | other | 13 | 26% |
| 21 | other | 12 | 24% |
| 28 | other | 3 | 6% |
| 0 | other | 2 | 4% |

## **S1 Figure – WGCNA Co-Expression Modules and KEGG's Biological Pathways Enrichment Heatmap for Subcutaneous Adipose**


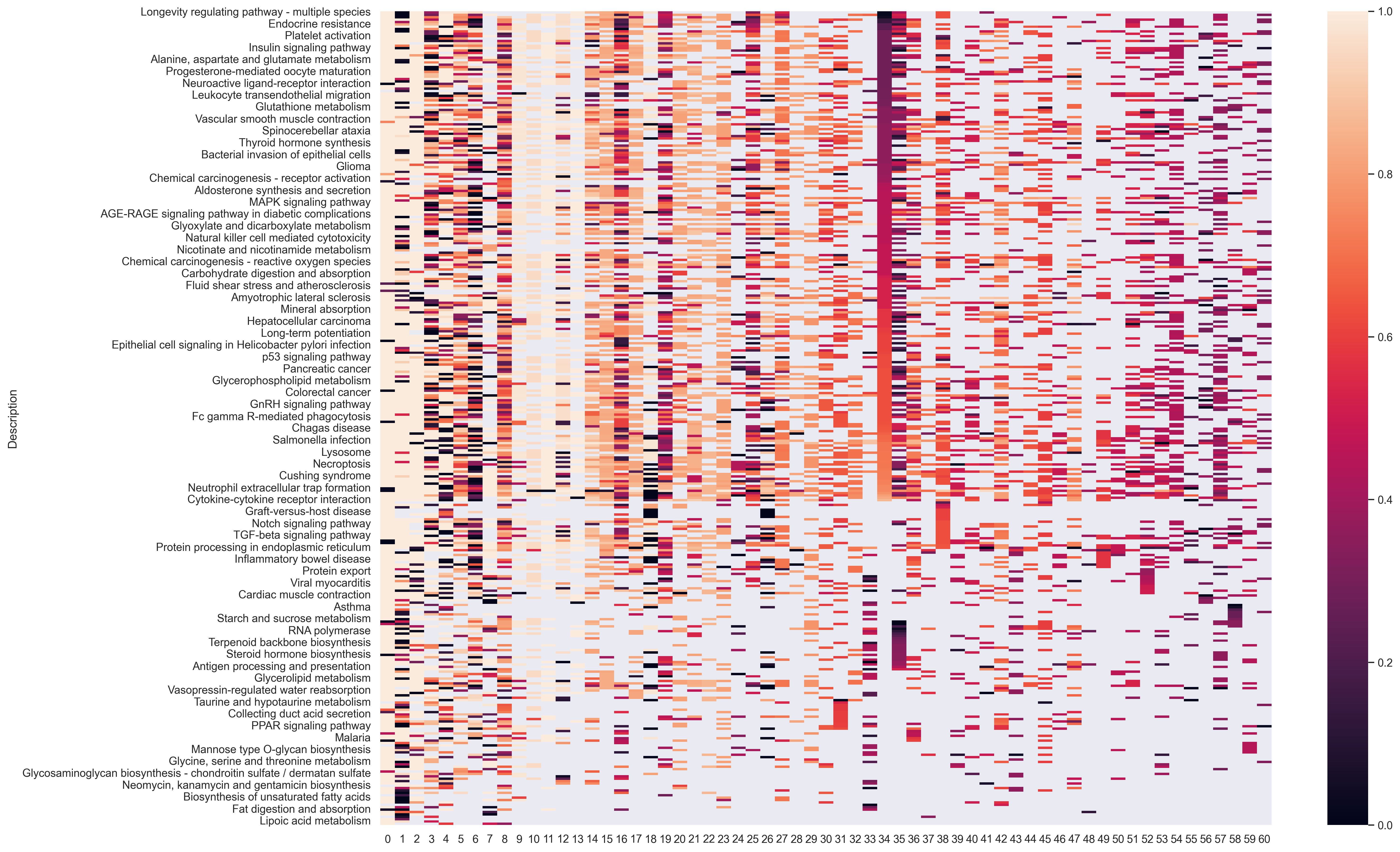


The heatmap above shows the p-values of the KEGG enrichment scores for each of the 60 modules created by the WGCNA algorithm for adipose subcutaneous.

## **S2 Figure – WGCNA Co-Expression Modules and KEGG's Biological Pathways Enrichment Heatmap for Visceral Adipose**


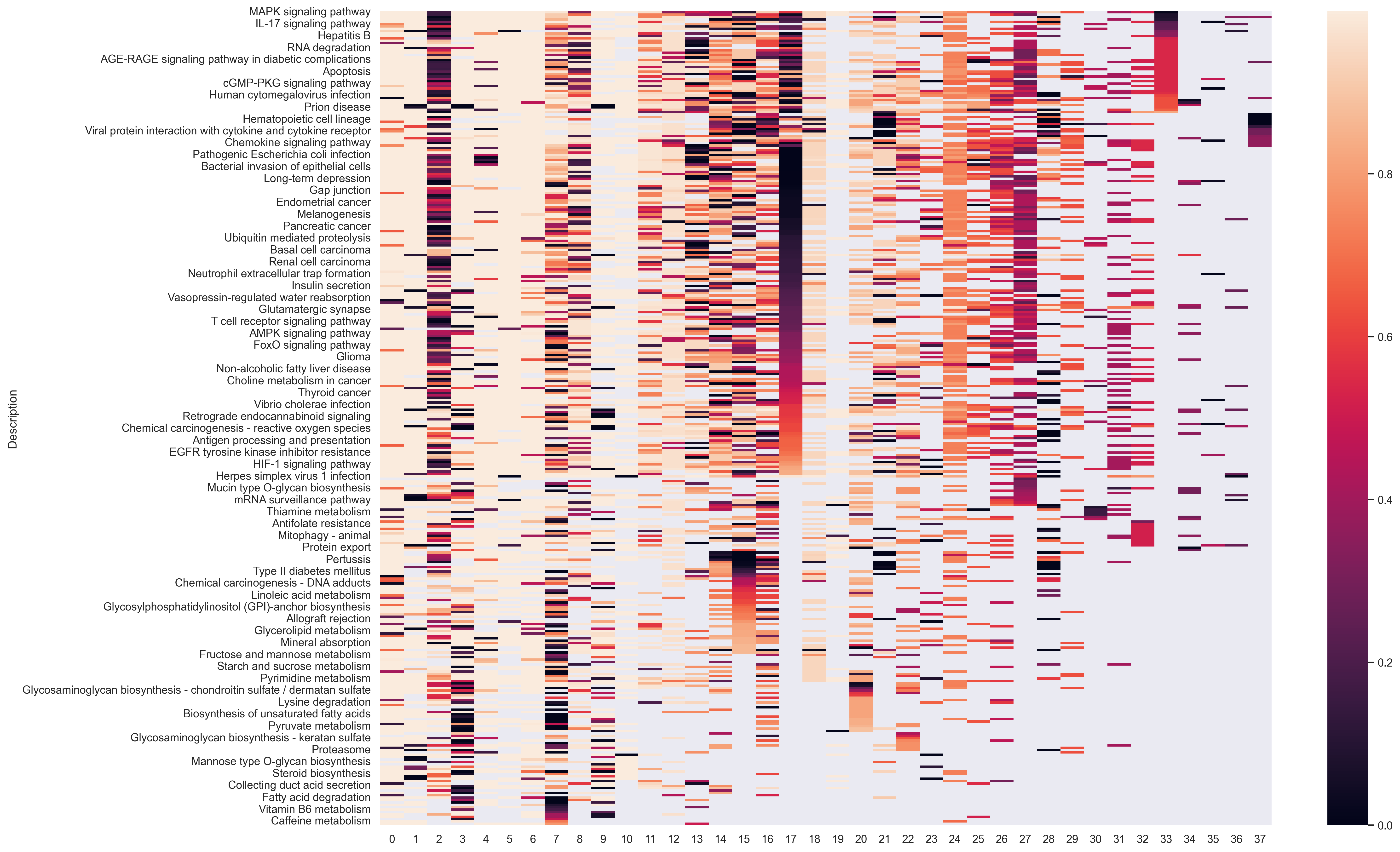


The heatmap above shows the p-values of the KEGG enrichment scores for each of the 37 modules created by the WGCNA algorithm for adipose visceral.

## **S3 Figure – WGCNA Co-Expression Modules Dendrogram for Subcutaneous Adipose**


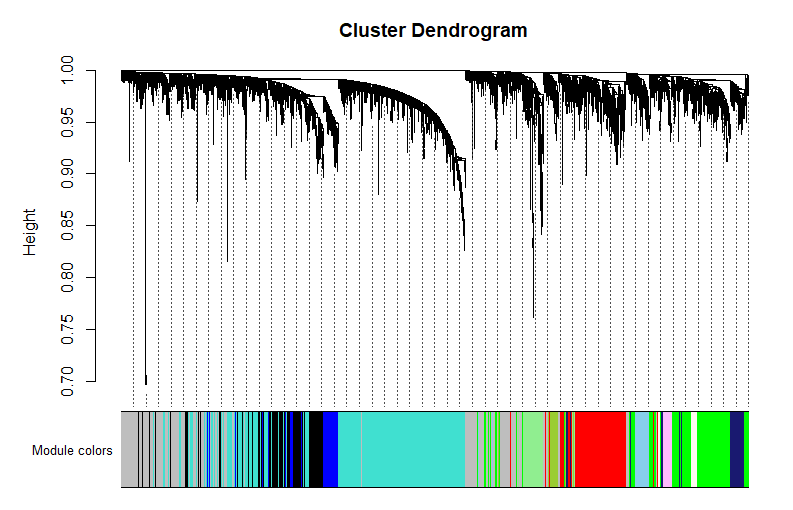


The figure above shows the clustering dendrogram of genes, with dissimilarity on the topological overlap, together with assigned module colors.

## **S4 Figure – WGCNA Co-Expression Modules Dendrogram for Visceral Adipose**

**
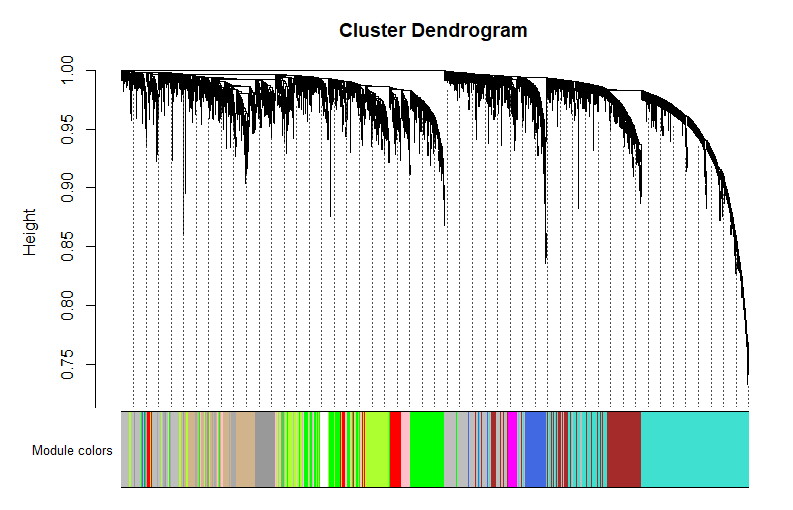
**

The figure above shows the clustering dendrogram of genes, with dissimilarity on the topological overlap, together with assigned module colors.

## **S5 Figure – SHAP value direction analysis for Subcutaneous Adipose**

The SHAP method can also illustrate the direction of each feature’s contribution to class classification. The figure shows the total of SHAP value magnitudes over all samples as a plot of features sorted in descending order by feature's relevance and uses SHAP values to highlight the distribution of feature impacts on model output prediction. This analysis can be generated for 2 types of classes (since it shows direction). Here we analyze and show how the value of the feature affects the metabolic class as opposed to the other classes. The horizontal position indicates the influence of each feature, i.e., whether that value's effect is related to a greater or lower prediction for the metabolic class. The coloring corresponds to each feature's original values across samples and indicates whether that feature value (pathway enrichment score) is high (in red) or low (in blue) for that observation. The SHAP values of each feature are represented on the x-axis and represent the feature’s impact on model output and the features (e.g., KEGG pathways) are on the y-axis. For example, a high value (red colored dots) of the enrichment score for the “Diabetic cardiomyopathy” KEGG pathway (the first feature from the top) has a negative impact (a negative SHAP value on the x-axis) on metabolic receptors prediction. I.e., a higher enrichment score for this pathway drives a metabolic prediction in most cases, increasing the probability of the sample being categorized as belonging to the metabolic receptors group.


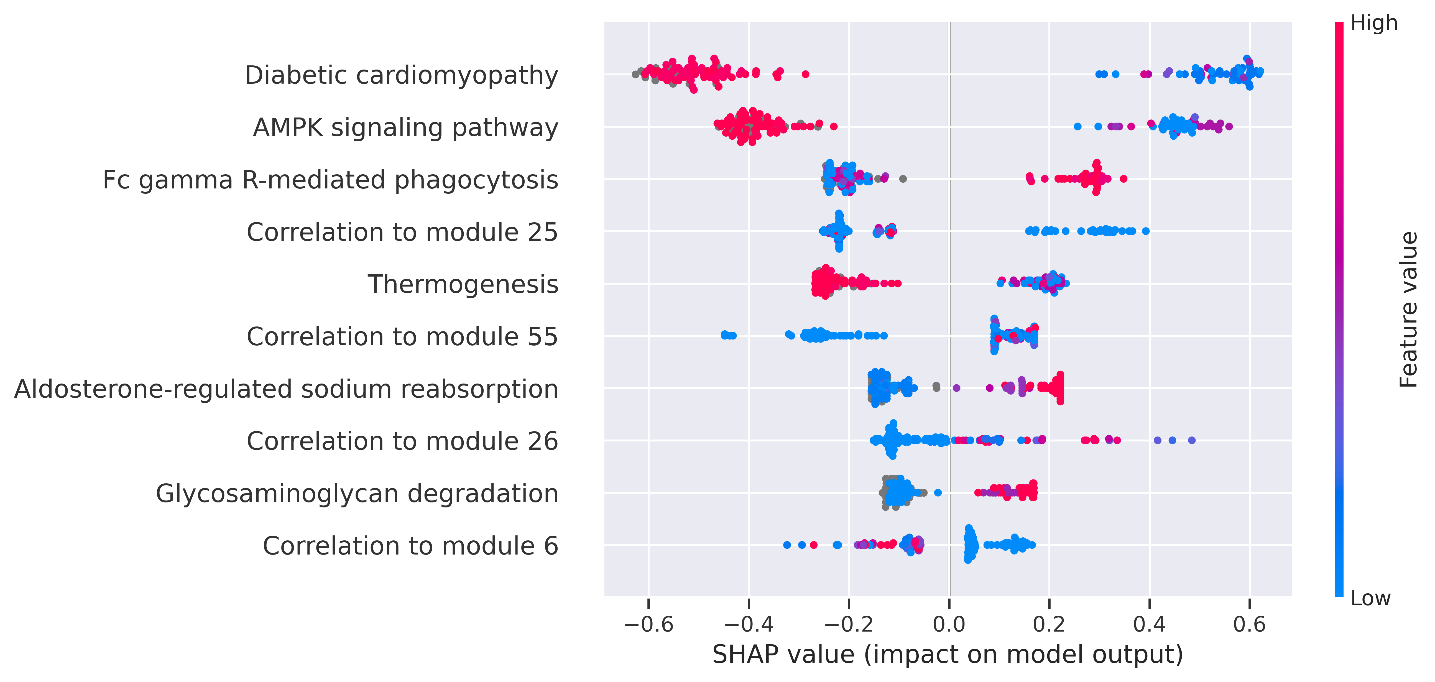


## **S6 Figure – SHAP value direction analysis for Visceral Adipose**


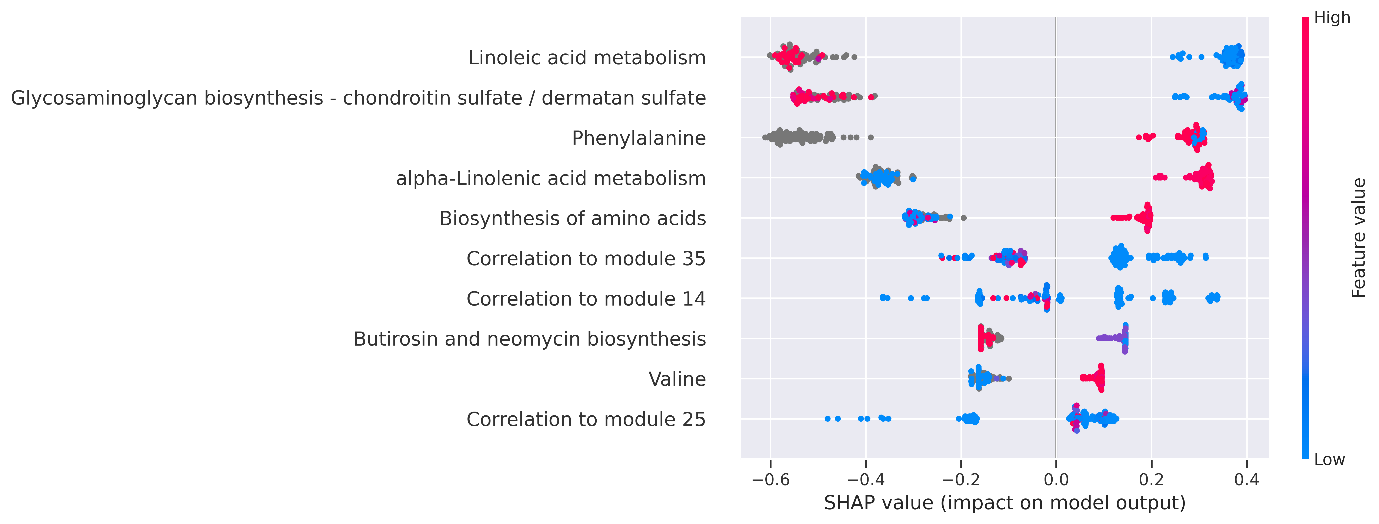


## **S7 Figure – Outliers removal using Isolation Forest**

An anomaly (i.e., an outlier) is an observation that significantly deviates from the other observations to arouse suspicion that it was generated by a different distribution. Anomalies, specifically in a big dataset, may follow very complicated patterns, which are difficult to visually detect in many cases. Anomaly detection is well suited for the application of Machine Learning techniques and the most common techniques employed for anomaly detection are based on the construction of a profile of what is “normal”: anomalies are reported as those instances in the dataset that do not conform to the normal profile. Isolation Forest uses a different approach: instead of trying to build a model of normal instances as in the common techniques, it explicitly isolates anomalous points in the dataset. Isolation Forest builds an ensemble of trees, then anomalies are those instances that have short average path lengths on the trees since they are "few and different", meaning they will require fewer random partitions of given attributes to be isolated. There are only two variables in this method: the number of trees to build and the sub-sampling size (number of samples in each tree). The main advantage of this approach is the possibility of exploiting the sub-sampling technique to an extent that is not feasible to the profile-based methods, creating a very fast algorithm with a low memory demand.


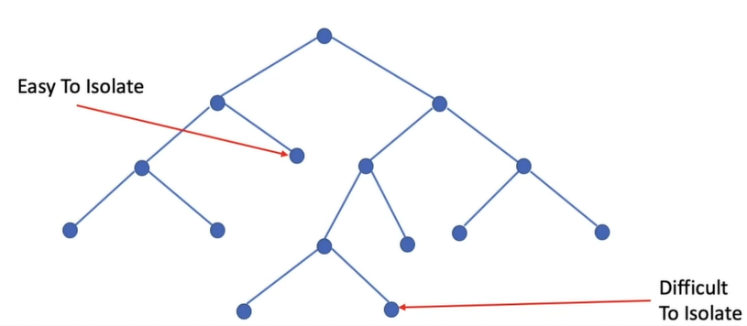


In the figure above, the sample in the lower leaf of the tree has a path length of 4 while the upper sample has a path length of 2. In that case, the upper sample is easier to isolate than the lower sample.

## **S8 Figure – WGCNA scale-free topology plots**


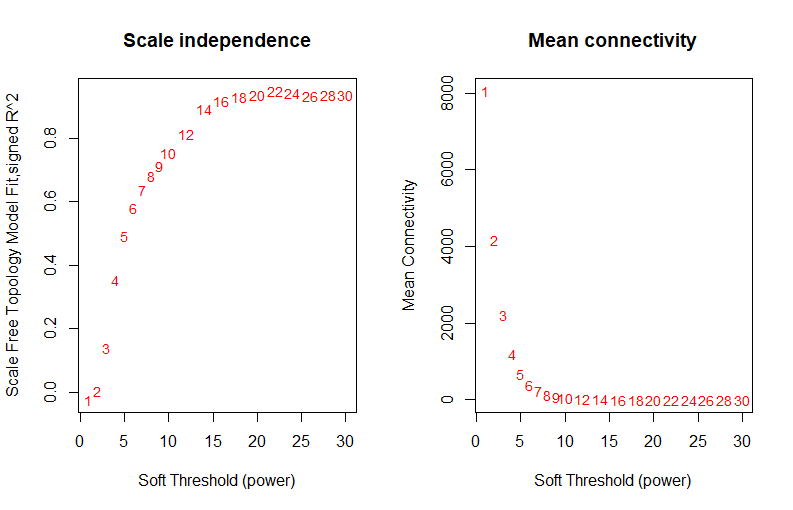


A B

Adipose Subcutaneous Scale Free Topology Plots

Analysis of network topology for various soft-thresholding powers. A. The scale-free fit
index (y-axis) as a function of the soft-thresholding power (x-axis). B. The mean connectivity
(degree, y-axis) as a function of the soft-thresholding power (x-axis)


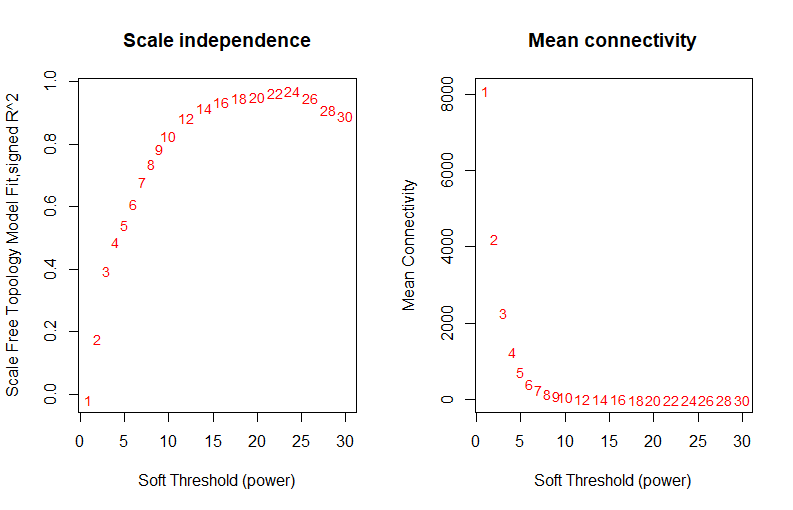


A B

Adipose - Visceral Scale Free Topology Plots

Analysis of network topology for various soft-thresholding powers. A. The scale-free fit
index (y-axis) as a function of the soft-thresholding power (x-axis). B. The mean connectivity
(degree, y-axis) as a function of the soft-thresholding power (x-axis)
